# Analysis of Antisense Expression by Whole Genome Tiling Microarrays and siRNAs Suggests Mis-Annotation of Arabidopsis Orphan Protein-Coding Genes

**DOI:** 10.1371/journal.pone.0010710

**Published:** 2010-05-26

**Authors:** Casey R. Richardson, Qing-Jun Luo, Viktoria Gontcharova, Ying-Wen Jiang, Manoj Samanta, Eunseog Youn, Christopher D. Rock

**Affiliations:** 1 Department of Biological Sciences, Texas Tech University, Lubbock, Texas, United States of America; 2 Department of Computer Science, Texas Tech University, Lubbock, Texas, United States of America; 3 Systemix Institute, Redmond, Washington, United States of America; East Carolina University, United States of America

## Abstract

**Background:**

MicroRNAs (miRNAs) and trans-acting small-interfering RNAs (tasi-RNAs) are small (20–22 nt long) RNAs (smRNAs) generated from hairpin secondary structures or antisense transcripts, respectively, that regulate gene expression by Watson-Crick pairing to a target mRNA and altering expression by mechanisms related to RNA interference. The high sequence homology of plant miRNAs to their targets has been the mainstay of miRNA prediction algorithms, which are limited in their predictive power for other kingdoms because miRNA complementarity is less conserved yet transitive processes (production of antisense smRNAs) are active in eukaryotes. We hypothesize that antisense transcription and associated smRNAs are biomarkers which can be computationally modeled for gene discovery.

**Principal Findings:**

We explored rice (*Oryza sativa*) sense and antisense gene expression in publicly available whole genome tiling array transcriptome data and sequenced smRNA libraries (as well as *C. elegans*) and found evidence of transitivity of *MIRNA* genes similar to that found in Arabidopsis. Statistical analysis of antisense transcript abundances, presence of antisense ESTs, and association with smRNAs suggests several hundred Arabidopsis ‘orphan’ hypothetical genes are non-coding RNAs. Consistent with this hypothesis, we found novel Arabidopsis homologues of some *MIRNA* genes on the antisense strand of previously annotated protein-coding genes. A Support Vector Machine (SVM) was applied using thermodynamic energy of binding plus novel expression features of sense/antisense transcription topology and siRNA abundances to build a prediction model of miRNA targets. The SVM when trained on targets could predict the “ancient” (deeply conserved) class of validated Arabidopsis *MIRNA* genes with an accuracy of 84%, and 76% for “new” rapidly-evolving *MIRNA* genes.

**Conclusions:**

Antisense and smRNA expression features and computational methods may identify novel *MIRNA* genes and other non-coding RNAs in plants and potentially other kingdoms, which can provide insight into antisense transcription, miRNA evolution, and post-transcriptional gene regulation.

## Introduction

Small RNAs (smRNAs), including microRNAs (miRNAs), endogenous small-interfering (siRNAs), and piwiRNAs are involved in transcriptional and post-transcriptional silencing pathways in plants and animals [1]–[5]. Their discovery has resulted in a paradigm shift: non-coding RNAs (ncRNAs) function as epigenetic regulators of transcription, splicing, export, stability, and translation superimposed on the Molecular Dogma. miRNAs are transcribed by RNA Polymerase II or III (Pol II or III) [6]–[8] and fold into characteristic stable hairpin secondary structures that are processed by Dicer enzyme complexes into mature 20–24 nucleotide (n.t.) sequences [5], [9]. After biogenesis and integration of the mature miRNA into the RNA Interference Silencing Complex (RISC), the miRNA acts as a specificity determinant by forming Watson- Crick pairs with the target mRNA molecule. The result is endonucleolytic cleavage and subsequent degradation of the message, translational inhibition, and/or transitive production of siRNAs by RNA interference-related mechanisms [5], [10]–[15].

Computational and experimental efforts in plants have explored long non-coding RNAs (ncRNA), RNA species with limited or no capacity to encode proteins [16]. Teramoto et al. first identified a *CR20* gene repressed by cytokinin, stress and/or developmental conditions in cucumber and a homolog in Arabidopsis that encodes no long ORFs [17]. A tyrosine kinase-like gene was found to have an antisense transcript, *ATH132404*, which does not appear to encode any protein [18]. Other ncRNAs were discovered by DNA library screening such as *ZCF83, RXF6*, and *RXW18*, in which *ZCF83* is antisense to a helix-loop-helix gene [19]. In 1999 and 2000, *At4* and its homolog, *AtIPS1*, from *IPS1*/*Mt4* family were shown to be induced by phosphate (P_i_) deprivation [20], [21]. *AtIPS1* inhibits the activity of P_i_ starvation-induced miR399 by a mechanism termed ‘target mimicry’ of base pairing without RISC cleavage [22]. Computational searches and experimental validation of expressed sequence tags (EST) have been the main focus for discovery of ncRNAs, from which hundreds of sequences have been identified [23]–[29]. Interestingly, miR162a and miR869 primary transcripts were originally described as ncRNAs, demonstrating the efficacy of finding miRNA-like sequences by characterizing ncRNAs.

Antisense transcription is a pervasive but poorly understood phenomenon associated with RNA interference and miRNAs in plants and animals. The function of miRNAs, their relationship to antisense transcripts, their subcellular pools, and the precise mechanisms by which these processes suppress gene expression remain elusive and controversial [30]–[33]. A homologue (ARS2) of the Arabidopsis zinc finger-containing protein SERRATE that functions in pre-mRNA splicing and miRNA processing has recently been shown to be a component of the nuclear RNA cap-binding complex in mice and to mediate both antiviral defense and developmental patterning in Drosophila [34], [35], establishing that RNAi- and miRNA-dependent processes are deeply conserved between plants and animals. Prokaryotes and simple eukaryotes have ncRNAs and antisense transcripts [36]–[39], but ncRNAs increasingly dominate the genomes of multicellular organisms as their complexity increases, in contrast to protein-coding genes [40]–[43], providing a plausible explanation for the “C-value paradox.” It is estimated that 40% of all transcription units in human and mouse genomes exist in cis-antisense co-expressed pairs [44], [45] and there is correlative evidence for a regulatory function of antisense in animals and plants [46], [47]. The 5′UTRs and first exons of genes with overlapping antisense transcripts are significantly longer than the genomic average, and a similar size distribution is observed for genes silenced by CpG island methylation in human cancer, supporting a role for antisense transcripts in regulation [48]. Recent results show that human genes are regulated transcriptionally by promoter-associated and terminator-associated antisense RNAs [49]–[53]. Studies of plant development and environmental stress responses have converged on the roles of ncRNAs and their metabolism as primary regulators of gene action, but it is still under debate to what extent those antisense transcripts are associated with siRNAs that couple exogenous signals to gene regulation [46], [54]–[64]. Understanding the origins of antisense ncRNAs may lead to new insights into fundamental processes such as tissue-specific and developmental gene regulation, chromatin dynamics, dsRNA biogenesis and processing, and genome evolution.

Plant miRNAs have high levels of complementarity to their target mRNAs, which greatly facilitates homology-based computational methods for *MIRNA* gene and target discovery in plants [65]–[76]. Nonetheless many recently discovered miRNAs and miRNA-associated smRNAs were instead uncovered functionally by deep sequencing of smRNA libraries [77]–[81]. We hypothesize that antisense transcription detected in plant whole genome tiling array transcriptome [82]–[84] and deeply sequenced smRNA datasets [85], [86] can serve as a biomarker to discover miRNAs and ncRNAs. Here we characterize *MIRNA* gene transitivity (antisense siRNAs mapping to miRNA hairpins) for Arabidopsis, *C. elegans* and rice and the topology (exon-intron signal-to-noise ratios) of strand-specific signals for annotated protein-coding genes. We identified several Arabidopsis *MIRNA* gene homologues and hundreds of potentially mis-annotated ncRNAs mapping to the antisense strand of annotated ‘unknown’ orphan protein-coding genes. A Support Vector Machine (SVM) was employed to analyze the importance of smRNA abundance features and sense/antisense topology as predictors of miRNA target sites and *MIRNA* genes. Our results suggest the utility of modeling whole genome tiling array transcriptome datasets for gene discovery and genome annotation.

## Materials and Methods

Perl [87] was used to extract, examine and manipulate data; scripts are available upon request. Before analysis of microarray data, those C-rich probes found previously to be affected by a sample amplification artifact [88] were removed. Other key programs were Unafold [89], SOAP (Short Oligonucleotide Alignment Program) [90], BLAST (Basic Local Alignment Search Tool) [91], and SVM for Matlab [92]. Unafold is based on dynamic programming principles of over-calculating the solution of thermodynamic free energy as a quicker solution than absolute calculation. Unafold's web-based portal is located at http://dinamelt.bioinfo.rpi.edu/quikfold.php. SOAP maps short probes from whole genome tiling arrays onto large databases of genomic DNA sequence more quickly than BLAST, but has a tradeoff of accuracy for speed. SOAP is not designed to work in conjunction with the Massively Parallel Signature Sequence (MPSS) smRNA databases [85], which is mostly comprised of 17 nt sequences where SOAP only performs at 50% accuracy. SVM for Matlab (MathWorks, Natick, Massachusetts; http://www.mathworks.com/products/matlab/) creates a prediction model to discriminate between training sets using supervised learning methods that require a set of features and a class label. Support vector machine (SVM) is an algorithm that learns by example to assign labels to samples [93]. SVMs have been successfully applied in various biological problem domains, particularly classification problems. Its popularity is due to its high generalization performance, sound mathematical foundation, and ease of use. The classification can be done on binary class data or multi-class data. Our dataset is of binary class. That is, the class labels are either +1 (target genes), or −1 (paralogs). SVM learns a prediction model from training samples. The model is used to predict an unseen sample's class label. The prediction model can be a decision line in two-dimensional data, a plane in three-dimensional data, or a hyperplane in higher-dimensional data. If a sample, or equivalently a point, lies above or on the decision plane it is predicted as ‘+1’ (target gene), or otherwise as ‘−1’ (paralog). SVM constructs a decision plane which lies furthest from the samples of both classes. That is why SVM classifier is called the maximum-margin hyperplane and this is the most distinguishing characteristic compared to other classification algorithms.

There are three main tasks in the workflow of establishing a classification model using an SVM that could have the ability to predict miRNA target binding sites: 1) identify useful attributes (features) for prediction and encode them into a dataset; 2) learn an SVM classification model; and 3) evaluate its performance. We identified and used three attributes to encode a candidate miRNA target gene: RNA transcript abundance from whole genome tiling microarrays; novel smRNA counts from deep sequencing of smRNA libraries; and free energy of binding of miRNAs to their cognate target genes.

### Expression data

The expression data is a novel feature for our classification system. We explored the antisense transcripts in relation to the miRNA targets by extracting the expression data for Arabidopsis miRNA target genes from existing high-resolution (25–36 b.p. probe size) whole-genome tiling micro-array datasets [82], [83]. All microarray data is MIAME compliant and has been extracted from NCBI Gene Expression Omnibus raw data series GSE601, 605, 636–639, and 2247. Graphical representation of the sense and antisense tiling array signals for all Arabidopsis and rice genomic sequences and AGI annotations can be viewed at www.systemix.org. The expression values for the sequences relative to the location in the genome were retrieved from the expression files using mapping coordinates built from the Arabidopsis Small RNA Project (ASRP) and Sanger mirBase databases [94], [95]. The results are compiled from five different biological samples, three of which correspond to different parts of the plant (flower, leaves, roots), and the other two from independent suspension-cultured dedifferentiated callus lines. Expression data corresponded to 800 base pairs (b.p.) surrounding the miRNA binding site on the miRNA target gene or *MIRNA* gene. These values were then normalized and were added at each position on all target genes to assess average effects.

The control (null) set for the miRNA target genes was created using homology-based bioinformatics searches to identify appropriate paralog “pseudo-target” genes from Arabidopsis. Paralogs are evolutionarily related to a miRNA target gene because of their high sequence homology except for the apparent lack of a remnant miRNA binding site. Paralogs were determined for the cognate genes by BLAST searches and manual inspection and assignment of “mock” miRNA binding site coordinates, and a file containing theoretical “binding site” locations was built. Using a similar approach as for the validated miRNA target sites, expression datasets were created with Perl scripts.

The tiling expression data [14] for the test sets (86 validated Arabidopsis miRNA targets representing 25 of 27 miRNA families; all *MIRNA* genes) and control (125 paralogs representing 16 families; Datafile S2) were normalized such that summation of expression values for a sample becomes 1 before they were applied to SVM. Expression signals at the locations ranging from 800 b.p. downstream and upstream relative to the miRNA binding site in target genes or miRNA* for *MIRNA* genes was stored into its own feature number at 25 b.p. resolution for the sense and antisense strand, resulting in 130 individual features for the general feature of expression levels.

### smRNA Counts

The second expression feature implemented for the SVM was smRNA counts mapping to the gene, based on previous results showing a statistically significant association of smRNAs with miRNA target genes [14]. In our analysis, the smRNA feature represented the number of expressed distinct signatures and their normalized abundance (transcripts per quarter-million reads, TPQ) obtained from MPSS and deep pyrosequencing datasets of different tissues and genotypes affected in smRNA metabolism, a conservative and quantitative method [85]. The list of potential miRNA target genes and *MIRNA* genes was processed by a bulk query of the MPSS [85] web portal (http://mpss.udel.edu). The normalized (TPQ) data was summed for unique reads (found only once in the genome) from multiple libraries. This treatment allows gene-by-gene comparisons of smRNA abundances. The MPSS dataset comprised four independent samples (FLR, inflorescence; RDR, *rdr2* mutant; two seedling libraries SD1 and SD2) that were used as separate features. The final feature was more qualitative than MPSS-based features: the sum of all normalized (TPQ) next-generation (454 pyrosequencing) smRNA datasets compiled from several different groups [78], [79], [94], [96], [97]. All Arabidopsis *MIRNA* genes that were listed as validated in ver. 11.0 of mirBase [95] were analyzed. The smRNA features were incorporated along side the expression levels. If there were no smRNA counts associated with a gene, the features were set to zeroes.

Antisense smRNAs with and without 5′ triphosphate moieties cloned from *C. elegans* somatic tissues [98] were BLASTed against mirBase stem-loops to map their topologies relative to mature miRNAs, miRNA*, and the hairpin loop.

### Thermodynamic Energy of Binding

The established method of quantifying miRNA complementarity to the target gene is accounted for in the thermodynamic energy of binding feature [99]. Target gene sequences were extracted and connected by a series of seven uridines to the matching miRNA sequence in order to create a “pseudo-hairpin” for reproducible folding. This string was analyzed by batch query of the UNAfold algorithm [89]. The ratio of the calculated free energy of miRNA: target to the free energy if the miRNA had perfect complementarity to the target gene (percent minimum free energy) was the final feature.

### Building a classification model

After building the dataset with the features consisting of expression data, smRNA counts and energy values for each of the possible target genes and the paralogs, ten-fold cross validation analysis was performed. The SVM was supplied training values for genes, actual target genes were given a positive class label (+1) and paralogs were given a negative class label (−1). The SVM developed a model based on the labels relative to the features and created a discriminant method using a linear kernel with default parameters to predict plausible target genes from paralogs.

## Results

### Evidence from smRNA and whole genome tiling array datasets for miRNA-associated transitivity

**Figure 1:**
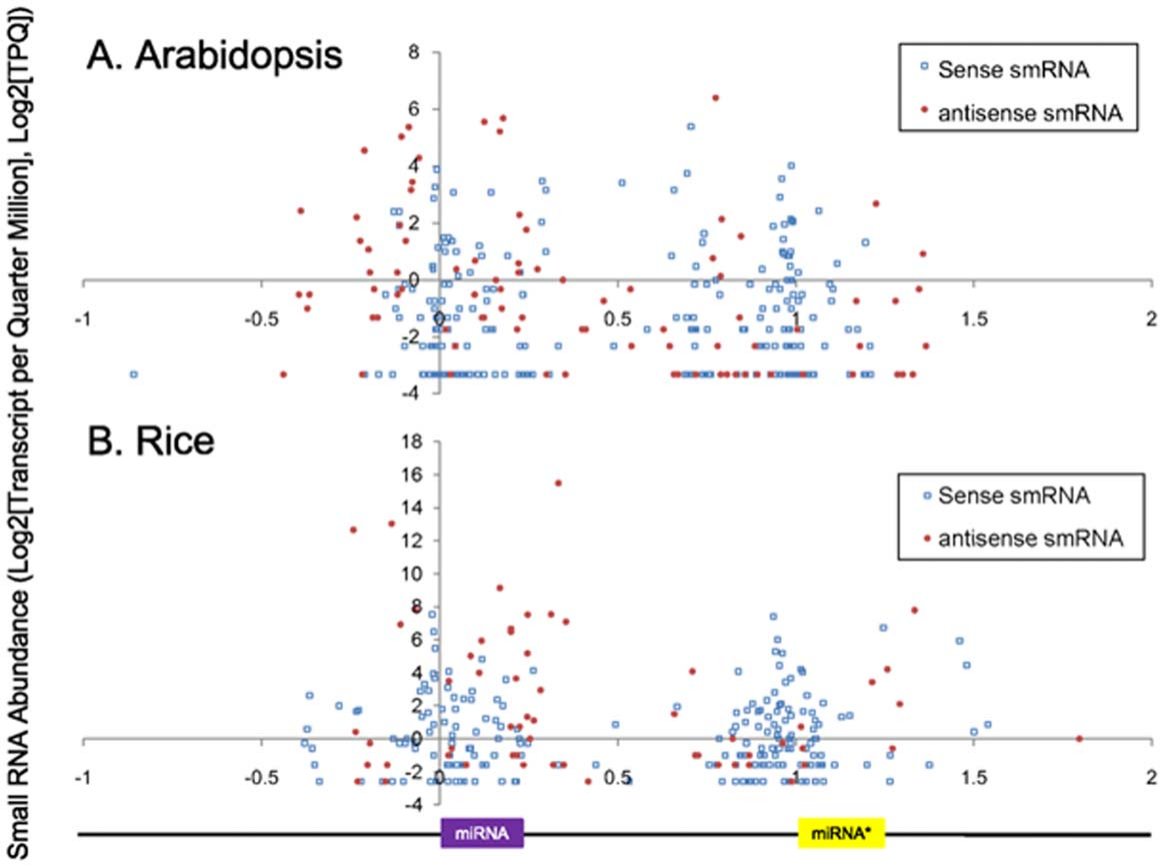
Abundance and topology of unique MPSS smRNA signatures with perfect matches to *MIRNA* hairpins. A) Arabidopsis *MIRNA* hairpins. B) Rice *MIRNA* hairpins. smRNA signatures were obtained from the MPSS Plus Database [85] (http://mpss.udel.edu) and searched against *MIRNA* hairpin sequences (http://microrna.sanger.ac.uk) and reference genome sequences (http://www.ncbi.nlm.nih.gov) by BLAST [91]. The normalized abundance of unique MPSS signatures (Log_2_, transcripts per quarter million [TPQ]) was plotted as a function of the normalized position of signatures relative to the start of the miRNA site on each individual hairpin. Sense smRNAs are indicated as open blue circles; antisense smRNAs are displayed as red closed circles. A cartoon for miRNA hairpin is shown under panel B to align the first nucleotide of mature miRNA (coordinate “0” on X-axis, purple box) and the first nucleotide of miRNA* (relative coordinate “1” on X-axis, yellow box) to the hairpin. See Datafile S1 for details.

In order to interpret antisense transcripts more broadly in a functional context, the datasets were qualified by characterization of signals associated with miRNAs [14] and ribosomal genes, the latter which serve as controls by virtue of being deeply conserved and highly expressed (see Supplemental Text File S1). Availability of whole genome tiling array and smRNA datasets for rice [84], [86] allow us to test the hypothesis that transitivity associated with miRNAs (antisense transcription leading to production of siRNAs that flank miRNA target sites or *MIRNA* loci) is broadly conserved in plants. Because there are few validated miRNA target genes in rice [100]–[102], we mapped and quantified unique rice smRNAs from deep sequencing datasets [86] to *MIRNA* hairpins available in miRBase [95] as a function of relative position to the mature miRNA and miRNA* and compared the topology to that of Arabidopsis siRNAs mapping to *MIRNA* hairpins (Figure 1; Datafile S1). Similar to our previous results [14] in Arabidopsis, abundant rice sense and antisense smRNAs were found for *MIRNA* genes (Figure 1B) with an apparent bias for 5′ upstream (relative to the sense strand) of the miRNA*. These data suggest activity of the miRNA (or miRNA*) binding to miRNA* (or miRNA) sites which triggers transitivity (spreading of siRNAs) in both directions on both strands. Recent results from *C. elegans* [98] have shown that two classes (26 n.t. 5′- monophosphate and 22 n.t. 5′-triphosphate species) of antisense siRNAs are produced against many transcripts in a two-step amplification by RNA-dependent RNA polymerases RRF-3 and RRF-1, respectively, in conjunction with DICER, Argonautes and other specificity determinants. We mapped 5′-monophosphate (primary) and 5′-triphosphate (secondary) antisense siRNAs to *C. elegans* miRNA hairpins and show in Supplemental Figure S1 (Datafile S6) that the most common primary and secondary siRNA map positions are the miRNA* and loop positions, respectively, similar to the observed topology of rice and Arabidopsis siRNAs in *MIRNA* genes (Figure 1).

**Figure 2:**
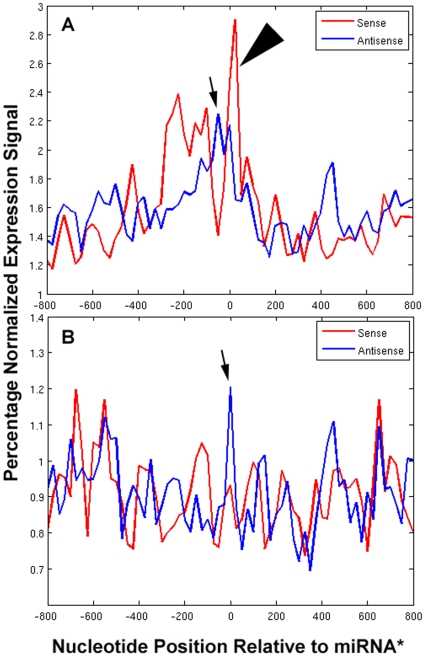
Normalized average percentage expression levels for 93 “ancient” (22 families) (A) and 68 recently evolved “new” (64 families) *MIRNA* genes (B), with miRNA* position as “0”. Sense strand is colored red and antisense blue. Note the abundant antisense signals mapping at or upstream to miRNA* sites (small arrow), and downstream sense signals for ancient *MIRNA* genes (large arrowhead) similar to miRNA target genes previously described [14]. See Datafile S2 for details.

We further mapped Arabidopsis whole genome tiling array sense and antisense transcript signals to 93 “ancient” *MIRNA* genes (those with at least one homolog in other distant plant species (27 families) and compared average normalized signal topology with 68 recently evolved “new” *MIRNA* genes (64 families) [77]–[79] by adding signals at each position of the data (Figure 2; Datafile S2). Ancient *MIRNA* genes had more abundant transcript signals on both sense and antisense strands, especially on the region of 200 n.t. upstream and downstream (relative to sense strand) of the miRNA* position (normalized expression > = 2.0, Figure 2A, arrows), whereas “new” *MIRNA* transcripts are not clearly evident above noise except for a peak signal precisely at the miRNA* position (normalized expression  = ∼1.2, Figure 2B, arrow). It is apparent that the ancient *MIRNA* genes have a ‘ping-pong-like’ expression topology (downstream sense, upstream antisense; Figure 2A, arrows) similar to that previously described for miRNA target mRNAs [14]. In order to extend the analysis to rice tiling array data, we analyzed whole genome tiling array signals for Arabidopsis and rice that had perfect matches to mature miRNAs, miRNAs*, siRNAs (17 nt reads from MPSS data [86]), and to probes mapping to other regions of the cognate hairpin. The results are shown in Table 1. Relative to the previously established signal cutoff of log_2_ >0.73 based on background signals from probes for both strands of promoters of ∼4,600 verified Arabidopsis genes [82], it is apparent that Arabidopsis *MIRNA* hairpin expression was low for most probes. Consistent with Figure 2A (upstream of miRNA* site), there was significantly more sense and antisense signals associated with miRNAs than elsewhere in the hairpins (Table 1; Datafile S3). For rice whole tiling array data there was higher signal associated with sense strand of miRNAs and antisense strand of miRNA* (Table 1), consistent with Arabidopsis data (Figure 2A), but the differences compared to other regions of the hairpin were not statistically significant and the rice tiling array data were not considered further. Supplemental Figures S2, S3, Supplemental Table S1, and Datafiles S4 and S5 document the quality of tiling array data by analyzing signal to noise ratios of ribosomal genes.

**Table 1:** Tiling array signals for all Arabidopsis and rice *MIRNA* hairpins^a^.

Species	Region of miRNA hairpin^b^											
	miRNA site			miRNA* site			other smRNAs			No smRNA		
	sense		anti	sense	anti		sense		anti	sense		anti
	(Log_2_Signal intensity)/probe											
	(number of probes)											
**Arabidopsis**	0.96^c^	1.26^c^		0.52		0.49		0.64	0.15		0.58	0.40
	(528)	(484)		(465)		(531)		(47)	(41)		(852)	(780)
**rice**	1.53	0.86		0.46		1.43		1.23	0.34		1.03	1.11
	(44)	(45)		(52)		(38)		(7)	(3)		(78)	(78)

a : Only the tiling array signals for regions of miRNA hairpins mapped by MPSS smRNA signatures will be counted. MPSS smRNA data was downloaded from http://mpss.udel.edu. See Datafile S3 for details.

b : Every probe is unique in the relative genome. A probe was counted as exclusively mapping to a region of the hairpin if a minimum of 11 contiguous n.t. in the probe overlapped with the 21 n.t. mature miRNA or miRNA* site, or 7 n.t. overlapped with the 17 n.t. MPSS smRNA signatures.

c : Significantly different than combined no smRNA signals, *P*<.0008 (Student's two tailed t-test, equal variance model).

### Assessment of Arabidopsis whole genome tiling array antisense signals

To characterize the quality of sense and antisense whole genome tiling array transcript signals for Arabidopsis, we endeavored to better understand the ‘topology’ of gene tiling array signals by calculating genome-wide the average exonic and intronic signal strengths for tiling array probes mapping uniquely to the sense strand of all 27,344 annotated protein-coding genes in TAIR9 and 50,090 rice genes (TIGR6.1, http://rice.plantbiology.msu.edu/, see Datafiles S4 and S5) that had tiling array [84] probe matches to exons. We divided average exon sense strand probe signals by the corresponding average antisense exonic signals to obtain a ratio of sense expression/antisense expression for each gene. We then sorted the genes (Datafiles S4 and S5) from lowest to highest based on this ratio, hypothesizing that highly expressed protein-coding genes (such as ribosomal protein genes) would be at the bottom of the ranking (sense expression >> antisense). Similarly we hypothesized that those validated miRNA target genes previously shown to produce abundant smRNAs and antisense transcripts in Arabidopsis [10], [12]–[14], [103] would be towards the top of the ranked genes because they produce more antisense transcripts (that in turn spawn antisense smRNAs). Table 2 presents results of an analysis of Arabidopsis ribosomal genes (Table 2, row 1) and miRNA target genes that produce abundant antisense siRNAs in Arabidopsis (row 2) as a function of ranking in the genome list for antisense exonic expression. As predicted from results of Supplemental Figure S2, there were ten times as many Arabidopsis ribosomal protein-coding genes in the bottom half of the antisense expression-ranked genome as the top half (Table 2; Datafile S4), but only 2.7 times as many rice ribosomal protein-coding genes (Supplemental Table S1, row 1; Datafile S5). For those select miRNA target genes in Arabidopsis known to produce antisense smRNAs, there were 3.3 times as many genes in the top half of the antisense transcript abundance genome list, which was statistically significant. No such relationship was observed for the validated and predicted homologues in rice (Supplemental Table S1, row 2).

**Table 2:** Antisense transcription signals relative to sense strand expression from Arabidopsis whole genome tiling arrays^a^.

Gene class	Genes with low sense/antisense exon signal ratio	Genes with high sense/antisense exon signal ratio	Ratio	*P* value^b^
**Ribosomal gene**	33	333	0.10	7.6e^−64^
**miRNA target^c^**	49	15	3.3	0.00002
**All “unknown” genes^d^**	3879	2943	1.3	0.000001
sORF^e^	426	210	2.0	3.7e^−18^
with as-TU^f^	692	598	1.16	0.005
qRT-PCR verified^g^	302	106	2.8	3.3e^−23^
**“unknown”(zero rating)^h^**	426	179	2.4	1.7e^−24^
sORF	280	142	2.0	8.6e^−12^
with as-TU	32	12	2.7	0.002
qRT-PCR verified	17	4	4.2	0.004
**“unknown” (one star)**	274	99	2.8	1.1e^−20^
sORF	119	50	2.4	5.7e^−8^
with as-TU	33	13	2.5	0.002
qRT-PCR verified	9	3	3.0	0.07
**“unknown” (one star) with EST**	362	102	3.6	3.4e^−36^
sORF	1	0	N.A.	N.A.
with as-TU	43	11	3.9	0.00001
qRT-PCR verified	44	18	2.4	0.007
**“unknown” (two stars)**	412	137	3.0	1.4e^−33^
sORF	2	0	N.A.	N.A.
with as-TU	74	26	2.8	0.000001
qRT-PCR verified	39	11	3.5	0.00007
**“unknown” (three stars)**	257	107	2.4	1.2e^−15^
sORF	8	4	2.0	0.19
with as-TU	39	19	2.0	0.006
qRT-PCR verified	27	4	6.7	0.00004
**“unknown” (four or five stars)**	2148	2319	0.9	0.005
sORF	16	15	1.1	1.0
with as-ncTU	471	517	0.9	0.07
qRT-PCR verified	166	66	2.5	0.000001
**All “unknown” with as-smRNA**	438	187	2.3	1.8e^−24^
with as-TU	56	31	1.8	0.005
qRT-PCR verified	53	10	5.3	0.000001
**Protein-coding with as-smRNA^i^**	982	720	1.4	0.000001
with as-TU	161	182	0.9	0.14
qRT-PCR verified	125	20	6.2	0.000001

**Table 2** **.** Footnotes.

a : Gene annotation is from TAIR Release 9 (http://www.arabidopsis.org/). Arabidopsis whole genome tiling array data was from previous reports [82], [83]. For each gene, the ratio of sense/antisense exon signal is calculated according to the following formula: ratio  =  [(sense exon signals/probe numbers)/(antiense exon signals/probe numbers)]/[(sense intron signals/probe numbers)/(antiense intron signals/probe numbers)]. See Supplemental Text File S1 and Datafile S4 for details.

b : One-tailed binomial distribution, normal approximation model, except as noted.

c : Validated and predicted miRNA targets were extracted from ASRP database for miRNAs 156, 162, 163, 168, 172, 393, 400, 403, 472, 773 and 780 (http://asrp.cgrb.oregonstate.edu). These targets produce significant numbers of antisense siRNAs [10], [12]–[14];.

d : Genes reported as “unknown” were collected from the TAIR9 release for Arabidopsis genome (http://www.arabidopsis.org).

e : Small open reading frames (sORFs) were from [27].

f : Genes with antisense transcript units were from [58].

g : Genes with antisense transcripts verified by quantitative RT-PCR were from Y. Xiao and C.D. Town, personal communication.

h : Unknown genes with different confidence ratings were from TAIR9 (http://www.arabidopsis.org). Zero rating means no expression data. One star rating means there is weak EST data, and/or another type of low quality functional evidence. Higher (2–5 star) rankings derive from qualitative meta-analysis of full-length cDNAs, proteomics, moncot and dicot cross-species sequence alignments, and genomic conservation.

i : Protein-coding genes with antisense smRNAs were from [107]; see Datafile S4.

### Arabidopsis antisense whole genome tiling array signals suggest some ‘unknown’ predicted proteins may be mis-annotated non-coding RNAs, including new *MIRNA* homologues

Rice and Arabidopsis genomes have been recently re-annotated based on multiple gene-finding and annotation algorithms that attribute confidence scores to exons based on different types of experimental and computational evidence [104], [105]. Taking advantage of the new features of the TAIR9 Arabidopsis genome release, we analyzed our genome-wide lists of Arabidopsis genes ranked as a function of relative antisense strand tiling array expression, focusing on annotated protein-coding genes defined as expression-confidence classes (star rankings) for Arabidopsis ‘unknown’ genes and ‘unknown’ expressed or ‘hypothetical’ genes (no expression data available) for rice. The results are summarized in Table 2 and Supplemental Table S1, respectively. For Arabidopsis it is evident that hundreds of ‘unknown’ genes with low expression confidence rankings (zero or one star) and those producing antisense smRNAs are significantly more abundant (about three-fold) in the top half of the genome ranked as a function of low sense/antisense exon signal ratio (Table 2). For those unknown Arabidopsis genes with high confidence expression data (four or five stars), the abundance ranking based on antisense expression is actually fewer in the upper versus lower halves of the genome, consistent with results for highly expressed ribosomal protein-coding genes (Table 2). The trend for more ‘unknown’ (including ‘expressed’) genes in the upper half of the rice genome ranked on antisense tiling array expression was barely discernable (Supplemental Table S1) and not statistically significant in the context of analogous Arabidopsis genes. We interpret this finding as consistent with the low quality antisense tiling array expression data for rice. A recent report described the antisense strand expression of some rice hypothetical genes [106].

The potential significance of results in Table 2 is that ‘orphan’ Arabidopsis genes predicted by gene-finding algorithms and having relatively abundant antisense strand expression may actually be ncRNA genes. We computationally tested this hypothesis five ways, and in addition found supporting expression evidence from ESTs (see below). The binomial distribution (upper vs. lower halves of the ranked transcriptome ratio of gene sense exon/antisense exon signals) of predicted unknown protein-coding genes that overlap recently published antisense ncRNAs [58] showed a similar pattern of two- to four-fold enrichment for TAIR9 expression confidence classes less than four stars (Table 2, rows “with as-TU”; Datafile S4). Two additional independent tests comprised the bionomial distributions for two exclusive sets comprising 1,044 predicted ‘unknown’ ORFs [27](Y. Xiao and C.D. Town, personal communication). Table 2 shows that for these two subsets of unknown genes (rows “sORFs” and “qRT-PCR verified”), similar to the genome-wide general pattern and the pattern for ncRNAs mapping to unknown genes, there were significantly more genes in the upper half of the ranked sense/antisense transcriptome for TAIR9 lower expression confidence rankings (star rating), especially for the qRT-PCR predictions (weighted mean of upper/lower ratio for zero-three star confidence  = 3.9, data not shown). As predicted by the working hypothesis that antisense transcripts are processed into smRNAs, genes that produce antisense siRNAs [107] were over-represented in the upper binomial distribution for exon antisense signal abundance, with strong evidence for over-representation of unknown protein-coding genes with independent ncRNAs mapping to them [58] or encoding unknown qRT-PCR tested genes (Y. Xiao and C.D. Town, personal communication) compared to all protein-coding genes (Table 2, compared bottom two rows).

A fourth computational test of the hypothesis was meta-analysis for congruence across the datasets, specifically whether 105 unknown genes recently predicted as small ORFs [27] or predicted and tested by conventional means (Y. Xiao and C.D. Town, personal communication) and having independent quantitative and qualitative evidence of strong antisense expression from tiling array experiments [58], [82], [83] clustered as a function of gene model quality. Results in Supplemental Figure S4 for 105 genes show a positive correlation (r = 0.61) between expression quality (TAIR9 star ranking) and measured abundance of antisense transcripts (sense exon/antisense exon signal ratio < unity), and an inverse correlation (r = 0.83) for expression quality rankings between recently predicted small unknown ORFs [27] and previously predicted unknown genes found by conventional algorithms (Y. Xiao and C.D. Town, personal communication). The latter class of genes is the subject of targeted expression studies by qRT-PCR and therefore is better represented in the two- to five-star expression rank classes.

A final computational test was to BLAST the Arabidopsis genome with known *MIRNA* hairpins to search for homologues, reasoning that some antisense transcripts may encode *MIRNA* homologues. Results are shown in Table 3 for candidate *MIRNA* gene homologues identified as mapping to the antisense strand of predicted protein coding genes and producing some antisense smRNAs. Consistent with our hypothesis, all of the *MIRNA* homologues were found in the upper half of the antisense expression-ranked genome list or on a previously described antisense non-coding RNA. For miRNA targets, vast majority of miR846 predicted and validated target genes (jacalin/lectin-like, which have extended homology to miR846 hairpin [78]) were also found in the top half of the list for higher expression of antisense transcription (Table 3). Three additional functional evidences supporting the results are that one predicted miRNA target, *AT1G07650*, which has a miR404 hairpin homologue on the antisense strand for its 3′-UTR, is up-regulated in the miRNA metabolism mutants *hst1-15* and *hyl1-2* [94], [97]. Another example is *AT4G03050/AOP3*, which has elevated expression in the *hen1-1* miRNA methyltransferase mutant and produces unique smRNAs sequenced from immuno-precipitated AGO4 complexes [96]. Two hairpin-containing genes, *At1G55045*, *At5g26262*, and a 7SL-like signal recognition particle ncRNA (*Ath-383*) [29] on the sense strand of *At2g31141* were uncovered by manual inspection of the predicted small ORF unknown genes for production of smRNAs predominantly from one strand (Table 3). *At1g55045* and *At5g26262* antisense transcripts can fold into stable hairpins (Supplemental Figure S5A; data not shown), produce moderately abundant phased 23–24 n.t. antisense siRNAs (Supplemental Figure S5B; data not shown), and the genes are methylated by DNA maintenance and de novo establishment methyltransferases MET1 [108] and DRM1-2/DRM2-2/CMT3-11 [109] respectively. *At5g26262* has significant homology (E<0.03) to a rice transposon ORSgTETNOOT00686 (http://plantrepeats.plantbiology.msu.edu/search.html) and seven other intergenic loci in the genome (data not shown). Because of the lack of a candidate miRNA/miRNA* duplex that maps to the *At1g55045* hairpin, this foldback does not meet the criteria of a miRNA [110] but may be a case of an evolving or devolving *MIRNA*-or *TAS*-like locus subject to transitivity [78], [111], [112] and processive cleavage by DICER-LIKE3/4 complexes.

**Table 3:** New miRNA homologs and hairpin-like sequences found on antisense strand of annotated protein coding genes^a^.

miRNA hairpin	Homologous genes with low sense/antisense exon signal ratio	Homologous genes with high sense/antisense exon signal ratio	TAIR9 annotation, position of homology	Expression data quality (star rating)^b^	Antisense EST?	E-value homology of AGI sequence to cognate hairpin
**miR156g**	AT2G19420		Unknown, intron	1		2 e^−54^
**miR404**	AT2G19300	None	Unknown, exon	5		3 e^−7^
	AT1G07650^c^	None	LRR-kinase, 3′UTR	4	AV529349	4 e^−11^
**miR414^h^**	AT1G68870	None	Unknown, exon	5		5 e^−8^
	AT2G21420	None	Zinc-finger like	5		2 e^−16^
**miR415**	AT1G74458	None	Unknown, exon	4		4 e^−24^
**miR783^h^**	AT1G66300	AT1G66290	F-box like, exon	2; 1		2 e^−34^; 3 e^−40^
	AT1G66310	AT1G66640		5; 1		7e^−24^; 1 e^−17^
		AT1G66320		1		2e^−21^
**miR824a**	AT4G24410		Unknown, exon	1	BX820858	1 e^−74^
**miR826**	AT4G03050^d^	None	AOP3^e^, exon	5		4 e^−10^
**miR841**	AT4G13570	None	HTA4^e^; intron/exon	3		2 e^−30^
**miR843**	antisense-TU Group4327^f^		Prmtr At3g48030			5 e^−9^
**miR846**	AT1G61230^g^ (including 11 candidate targets)^g^	(including 2 candidate targets)^g^	jacalin-like, exon	2		9 e^−19^
**miR855^h^**	AT2G06095	None	Unknown, exon	2	EG435138	5 e^−32^
**Hairpins**	AT1G55045		Unknown, exon	0	phased	0.03^i^
		AT5G26262		0	smRNAs	
**7SL-like ncRNA^j^**		AT2G31141	Unknown, exon	5	smRNAs	3 e^−20^

**Table 3** **.** Footnotes.

a : Gene annotation was from TAIR Release 9. For each gene, the ratio of sense/antisense exon signal is calculated according to the following formula: ratio  =  [(sense exon signals/probe numbers)/(antiense exon signals/probe numbers)]/[(sense intron signals/probe numbers)/(antiense intron signals/probe numbers)]. All Arabidopsis genes were ranked based on this sense/antisense exon signal ratio. See Supplemental Text File S1 and Datafile S4 for details. All listed genes produce antisense smRNAs except for AT1G68870 which has a sense smRNA [78], [85], [94]. AT1G74458 encodes miR415 homologue on the sense strand. See http://mpss.udel.edu.

b : The star rating for gene expression refers to the legend of Table 2.

c : expression elevated in miRNA metabolism mutants *hst-15* and *hyl1-2* [94]. AT1G07650 was previously predicted as a target of miR404 [159].

d : expression elevated in a miRNA metabolism mutants, *hen1-1* [94].

e : Homologues *AT4G03060*/*AOP2*/and *AT2G38810*/*HTA8* were previously described as evolutionarily-related loci for miR826 and miR841, respectively [78]. Interestingly, *AT4G03050*/*AOP3* is a source of smRNAs sequenced from immuno-precipitated AGO4 [96].

f : A 2.2 kb antisense non-coding RNA described by Matsui et al. [58] that overlaps with At3g48030 and its promoter.

g : validated and predicted jacalin/lectin targets [77], [78], [94]. Genes with low sense/antisense exon signal ratio: AT1G52050, AT1G52060, AT5G28520, AT1G52120; AT1G52130, AT1G60130, AT5G38550, AT5G49870, AT5G49850, AT1G57570, AT1G60110; Genes with high sense/antisense exon signal ratio: AT2G25980, AT1G52070.

h : There is bioinformatic evidence these are not bona fide miRNAs: miR414 is homologous to transposon ATHAT1 and rice ORSgTETN00400025 (E  = 2 e−10) [105] (http://plantrepeats.plantbiology.msu.edu/search.html); miR783 is homologous to AT1G46120 transposable element gene (E  = 3 e−57) and maps between predicted F-box-like homologues AT1G66300 and AT1G66331; miR855 has significant homology to antisense strand of miR401 (E =  2e−37; noted also in [65]), VANDAL17, and Gypsy_Ty3-like transposons (E =  1e−108).

i : Significant homology to unclassified rice transposon ORSgTETNOOT00686 (http://plantrepeats.plantbiology.msu.edu/search.html).

j : 7SL is the ncRNA component of the signal recognition particle involved in targeting and translocation of proteins to the endoplasmic reticulum. There are three 7SL homologues described in Arabidopsis; AT2G31141 produces abundant smRNAs from the sense strand and was previously described as *Ath-383* 7SL-like ncRNA [29].

Manual inspection of ESTs associated with unknown genes that produce abundant antisense whole tiling array signals (average sense exon/antisense ratio < = 0.5) found 33 documented cases of antisense ESTs and 65 pairs of overlapping genes forming nat-*cis*-antisense transcripts [46], [56]. In addition, 11 predicted small unknown ORFs map immediately adjacent and on the same strand as neighboring genes, which suggests these transcripts represent unannotated 5′ and 3′ exons. Supplemental Table S2 lists the genes and their expression features, which represent all subclasses in Table 2 including genes with high confidence TAIR9 expression rankings (four and five star).

### Sense/Antisense tiling array transcriptome topology and smRNA abundance as expression-based features of Support Vector Machines (SVM) for *MIRNA* gene/target prediction

Machine learning algorithms for *MIRNA* gene and target site prediction utilize sequence complementarity as the primary feature, which is tractable in plants due to extensive homology between miRNAs and their targets but of limited use in animals [75]. Because plant and animal miRNA pathways share mechanisms and components with RNA interference and post-transcriptional gene silencing, we hypothesized that antisense expression-based topological features may be a useful predictor of miRNA targets and *MIRNA* genes. A molecular mechanism has been established [80], [113], [114] for the observed abundant downstream sense strand tiling array signal of miRNA target genes in which 3′ exonuclease degradation of the upstream cleavage product by EXORIBONUCLEASE4 (XRN4) is postulated. However the mechanism resulting in upstream antisense signal for target genes [14] and *MIRNA* genes (Figure 2A) has yet to be elucidated. The downstream-sense/upstream antisense Arabidopsis whole genome tiling expression data for validated miRNA target genes versus non-target paralogs was adopted as a key expression feature, along with smRNA abundances and thermodynamic energy of binding to implement an SVM for prediction of miRNA targets and genes. The normalized tiling array expression signals from 800 base pairs upstream and downstream on the sense and antisense strand, respectively, of miRNA binding sites [14] was extracted. This resulted in 130 values associated with each of the genes in the training set. The next feature consisted of smRNA counts and resulted in five additional features for the SVM. Four of the features correspond to the MPSS data from four different Arabidopsis samples: flowers, *RNA-dependent RNA Polymerase 2* (*rdr2*) mutant, and two seedling libraries [79]. The fifth smRNA feature corresponded to the sum of all normalized (TPQ) unique smRNA reads mapping to loci from many pyrosequencing experiments [78], [79], [94], [96], [97] (Datafile S2). The last feature was that of sequence complementarity represented mathematically as relative thermodynamic stability. The most stable combinations of miRNA and the target gene or *MIRNA* genes were normalized to percent minimum free energy which works well for plant miRNAs because plants possess near perfect complementarity between miRNA and target genes [65], [115].

In machine learning such as SVM where the goal is to classify samples, the “Gold Standard” refers to a set of data that can be used to train the prediction model and to test predictions. Our dataset was based on validated miRNA targets including the previously documented cases of transitivity (i.e. *PPR* and *AGO1* genes [10], [13]). We used various measures of SVM classification performance to evaluate the individual features, assigning validated Arabidopsis target genes the value of unity and the negative control paralogs [14] the value of negative one. The dataset was then analyzed through ten-fold cross validation. The various combinations of features were analyzed to evaluate the importance of each in identifying correctly the validated miRNA target genes.

#### Accuracy, Specificity, Sensitivity, and Precision

The accuracy was calculated for the ten-fold cross validation performed on the dataset. Table 4 lists the results for each feature alone and in combination for this statistic. The smRNA counts and tiling array expression topology alone and in combination were weakly predictive (∼60–70%), but not robust compared to the biological standard of thermodynamic stability (97%; Table 4). These results suggest the expression features under study may be useful features, but additional specificity determinants must be identified to strengthen an SVM for miRNA target gene prediction based primarily on expression. Table 4 also reports further statistical analyses of SVM specificity, sensitivity and precision. Specificity refers to how well a classification test can identify the negative cases, namely the probability to classify a gene as −1 if the target gene is a paralog with no miRNA binding site. All three features performed well for specificity 96–100%). The sensitivity of SVM is an evaluation of the test to predict the targets (+1 class). smRNAs were a weakly sensitive feature, and expression topology was insensitive as a predictor of miRNA targets (Table 4). The Positive Predictive Value, or the precision, addresses the evaluation of the machine. The number represents the probability that if the SVM predicted the gene to be a target, how likely is it a bona fide target gene. This test is the reverse of the previous two; the sensitivity and specificity test the machine in the respect of if the actual label is known, how likely is it to identify it correctly. smRNAs were a fairly good (87%) feature of SVM performance, but expression topology was not (Table 4).

**Table 4:** Accuracy, Sensitivity, Precision and Specificity of an expression-based Support Vector Machine for miRNA target gene prediction trained on 86 Arabidopsis miRNA target genes and 125 non-target paralogs.

Combination	Accuracy	Sensitivity	Precision	Specificity
**Expression Levels, smRNA Counts, Energy**	0.972	0.977	0.955	0.968
**smRNA Counts, Energy**	0.972	0.977	0.955	0.968
**Expression Levels, Energy**	0.972	1.000	0.935	0.952
**Expression Levels, smRNA Counts**	0.697	0.314	0.844	0.960
**smRNA Counts**	0.697	0.302	0.867	0.968
**Energy**	0.970	1.000	0.945	0.960
**Expression Levels**	0.592	0.000	NaN^a^	1.000

a : NaN: Not A Number, due to division by zero.

See Datafile S2 for details.

#### Further SVM testing on *MIRNA* genes

*MIRNA* genes are transcribed by RNAPol II [7], [8], [116] and therefore polyadenylated *MIRNA* precursor gene transcripts should be detected in the whole genome tiling microarray datasets and evidence (Figure 2) supports this model. Analysis of smRNA abundances and map positions on the antisense strand of *MIRNA* genes (which are generated by transitive processes) established that *MIRNA* genes, viz. at the complementary “miRNA*” position of the foldback, produce phased antisense siRNAs in a process similar to the working model of miRNA target genes that produce siRNAs [14](Figure 1A). Using the analogous features of the miRNA target genes and paralogs, the SVM was implemented on the miRNA* dataset in order to examine its utility for predicting *MIRNA* genes, since they are transcribed similarly and have complementarity at the miRNA* position to mature miRNAs (and thus homology to miRNA target genes). To facilitate SVM evaluation, the miRNA* were labeled as +1, assuming the sequences would exhibit properties of miRNA targets. Table 5 displays results of the SVM evaluations. Using normalized expression topology, energy of binding, and the sum of sense and antisense smRNA reads mapping to regions of the hairpin other than mature miRNA or miRNA*, the SVM produced results nearly as predictive (81% versus 97%) as the Gold Standard training set of miRNA target genes (compare Tables 4 and 5). The comparison of ancient versus new *MIRNA* gene predictions by the SVM was consistent with the expression topologies; the ancient miRNA*s display the “ping-pong” topology (Figure 2A) analogous to that seen in the miRNA target genes [14] and produced the better result (84%) from the SVM analysis (Table 5).

**Table 5:** Accuracy of the Support Vector Machine in predicting Arabidopsis *MIRNA* genes based on energy, expression topology and smRNAs.

Test	Accuracy of Prediction
**“93 Ancient” *MIRNA* genes**	0.841
**“88 Newly-evolved” *MIRNA* genes**	0.765
**Total miRNA**	0.808

See Datafile S2 for details.

## Discussion

### Antisense transcripts detected by whole genome tiling arrays are real

Our analyses [14] and those of others [117], [118] establish by multiple independent criteria that Arabidopsis antisense transcripts are real and of biological significance. Results from different whole genome tiling array technologies and platforms have shown congruence (e.g. Supplemental Table S2) for many antisense transcripts [58], [82], [83]. We have shown that *MIRNA* genes from both Arabidopsis and rice produce antisense smRNAs ‘spreading’ from the miRNA and miRNA* sites. Consistent with Figure 2A (upstream of miRNA* site), there was significantly more sense and antisense signals associated with miRNAs than elsewhere in the hairpins (Table 1; Datafile S3). Functional evidence of the antisense transcripts is seen by statistically significant over-representation in the upper half of the Arabidopsis transcriptome ranked on exon sense/antisense signal abundance for all genes including well-annotated protein-coding genes that produce antisense siRNAs (Table 2, last row). An additional evidence is strong over-representation in the upper half binomial distribution of unknown protein-coding genes with independent ncRNAs mapping to them [58] (Table 2, “with as-TU” rows). The breadth of extant antisense EST coverage (Supplemental Table S2) which includes genes with high confidence TAIR9 expression rankings (four and five star) is prima facie evidence that antisense transcripts identified by whole genome tiling arrays are biologically significant and support our computational evidence that a significant number of unknown predicted protein-coding genes are actually ncRNAs. The extant Arabidopsis tiling array data quality is high, but that of rice is not (Supplemental Figures S2, S3). This situation is likely due to the lower number of probes with perfect matches between the *Oryza sativa* var. indica reference genome (from which the tiling array probes were designed) and the *MIRNA* hairpin sequences in miRBase which have been sequenced predominantly from japonica (Nipponbare) varieties. A recent report describes antisense transcripts and siRNAs associated with hypothetical genes in rice [106], consistent with our results (Supplemental Table S1; Datafile S5).

### Expression-based computation as a means to ncRNA discovery and genome annotation

In this study we approach the broad question of applying computation to deep experimental expression datasets to develop methods for gene discovery, focusing on miRNAs, ncRNAs, and antisense transcripts. Because these RNA classes span eukaryotic kingdoms where the molecular processes are deeply conserved but the molecules themselves are not, genomic analysis of antisense expression patterns in plants may reveal associations (a la ‘a smoking gun’) that can provide insight into animal miRNA and ncRNAs, where complementarity is less conserved. Analogous approaches for miRNA target genes classified according to the promoter features of the cognate *MIRNA* genes have been described [119], [120]. We show that the phenomenon of spreading/transitivity of smRNAs associated with miRNA target genes and *MIRNA* genes in Arabidopsis is conserved in rice (Figure 1). Similar processes occur on worm *MIRNA* gene transcripts (Supplemental Figure S1), likely mediated by multiple interactions between RNA-dependent RNA polymerases RRF-1 and RRF-3 and associated with DICER and Argonautes NRDE-3 and ERGO-1 [98]. It is interesting to note that those *MIRNA* genes (e.g. miR158, miR159/319, miR164, miR167, miR168, miR172) whose transcripts accumulate in post-transcriptional processing mutants [80], [114], [116] also produce abundant smRNAs (Datafile S2)[14]. Xue et al. also observed antisense smRNAs associated with several rice miRNAs and miRNA*s [121] and noted an example (miR55) from *C. elegans*. Taken together with reports of a novel RNA-dependent RNA polymerase in Drosophila [122] and functional antisense miRNAs in Drosophila and mouse [123]–[125], our data in rice, *C. elegans*, and Arabidopsis [14] lend credence to the notion that similarly complex transitivity mechanisms operate on plant and animal miRNAs. We show that antisense transcription signals for *MIRNA* and protein-coding genes are detectable by whole genome tiling arrays (Figure 2; Supplemental Figures S2, S3; Table 1), providing evidence of the molecular mechanism of smRNA production. However, the low abundance of the antisense signals requires high quality microarray data (Figure 2; Supplemental Figures S2, S3) that are not yet available for rice (Supplemental Figure S2, Supplemental Table S1). Deep sequencing of mRNAs and epigenetic marks on DNA reveal hidden facets of RNA processing, chromatin remodeling, and gene regulation, but the method is expensive. Computational analysis of smRNA datasets, which are less costly on a molar basis to generate, in conjunction with inexpensive high resolution custom tiling microarrays can provide a more integrated view of gene expression, especially in genomes with limited annotation.

We incorporated expression data, smRNA counts, and thermodynamic energy of binding as features for a Support Vector Machine to build a model for prediction of miRNA target sites in Arabidopsis. Using a dataset based on validated Arabidopsis miRNA targets, the machine was internally tested based on accuracy, precision, sensitive and specificity. The results were modestly supportive of a predictive value for smRNA counts over tiling array expression signals, suggesting both these features have potential utility as filters in miRNA prediction methods over thermodynamic stability alone. The performance of the SVM was further tested with an external dataset: *MIRNA* genes. Although the miRNAs* corresponding to ancient miRNAs produced more supportive results, the newly discovered miRNAs* were also fairly predicted (Table 5). Mathematically modeling the ‘downstream sense, upstream antisense’ tiling array signal to describe more precisely the transitive activity and reduce the dimensionality might improve the performance of the SVM. The extreme case is seen when considering only those probes with perfect matches to miRNA hairpin domains (Table 1), which decreases noise and feature dimensions of the machine. Improvements such as utilizing a non- linear kernel and optimizing the “slack” parameter may improve the power of the SVM. The machine can be further developed with datasets of predicted miRNA targets [65]–[70], as well as candidate *MIRNA* genes and targets from purely computational methods. Thousands of predicted miRNAs in Arabidopsis and rice have no functional evidence to support their being expressed or having bona fide targets, and therefore represent a large investment to qualify by wet lab methods. The SVM could facilitate prioritizing those that have a greater likelihood of being real based on collective expression topologies. However, our results that qualify the rice tiling array expression data as low-quality (Table 1, Supplemental Figure S2, Supplemental Table S1) limit the potential of an expression-based SVM for rice until a high-quality tiling expression dataset is available. Optimizing the SVM features based on biology (e.g. sizes of the siRNAs, “phasing”) are other candidate features for adoption. Two new miRNA families (miR2118, miR2775) conserved in monocots (and *Phaseolus* for miR2118 [126]) were recently discovered by analysis of rice phased siRNAs produced from TASi-like target ncRNAs [127].

Gene models suffer from errors in reading frame, exon border definition, and exon identification. It is estimated that 13% of the Arabidopsis proteome is incomplete due to approximately equal numbers of missing and incorrect gene models [128], [129], suggesting that there is ample scope for gene discovery even in well-annotated genomes. Whole-genome tiling arrays have utility for characterizing alternative splicing [130]. Tiling array expression and TAIR9 confidence rankings are useful metrics for ncRNA discovery (Supplemental Figure S3, Supplemental Table S2). Our computational results begin to address expression ‘topology,’ the relationship between RNA expression signals and gene structure. We show, in the case of exons versus introns, that expression topology is a valuable metric for interrogating genome annotation. Arabidopsis signals show excellent congruence with exon/intron annotations in all five samples (from two different technology platforms) with only a slight bias of expression signal toward the 3′ end of the gene and minimal signal in the 3′-UTRs (Supplemental Figure S2; Datafile S4). This aspect of gene expression topology can be developed further by calculating an integral for each separate functional domain of a gene (promoter, 5′ UTR, exons, introns, 3′ UTR) and modeling expression topology to identify outliers that could facilitate gene discovery and genome annotation. Several groups have recently published Arabidopsis whole genome tiling array transcriptome studies on stress responses [58], [119], [120], [131] and note changes in 5′ and 3′ UTR and *MIRNA* gene expression. The existence of promoter-associated antisense transcripts in animals that regulate transcriptional activation and repression by RNAi-associated processes [49], [50], [132]–[137] suggest that tiling array interrogation of promoter-associated RNAs can indentify similar classes of ncRNAs in plants.

Applying machine learning algorithms, we could identify associations between miRNA target genes or *MIRNA* genes and smRNAs (Tables 4, 5) and between protein-coding genes and ncRNAs (Supplemental Table S2) that fit a model of transitivity based on their antisense expression topology. Our methods reveal the potential of expression-based machine learning and unsupervised association to discover new miRNAs, target genes and ncRNAs based on expression features such as strand bias for production of phased siRNAs (Supplemental Figure S5). Contingent upon generation and availability of high quality datasets, whole genome tiling array transcriptomes and deep smRNA datasets such as for rice [85], [86] and other species will be suitable subjects for further computational methods testing and analysis. Expression-based determinants have potential applications for ncRNA discovery in other kingdoms and species where miRNA binding site free energies are lower, especially in transitive processes which are poorly understood.

The method presented is equally applicable to transcriptome data generated from ultra high-throughput sequencing (UHTS) approaches. Conceptually, short read data can be represented in a format similar to tiling array data (genomic location versus read frequency instead of array signal). The added benefit of UHTS is that signals for any genomic coordinate are potentially generated rather than fixed a priori with predetermined probes in tiling array experiments. Therefore, UHTS data is conceptually equivalent to tiling array data with probes derived from every nucleotide of the genome- once the transcriptome data is converted to genomic coordinate versus signal representation, the methods described can follow without change.

The presented approach is applicable to animal systems. *C. elegans* and *D. melanogaster* are likely to be the best candidates to directly apply this approach, because their genomes are well annotated with whole genome tiling array and EST data [138]–[140]. Moreover, their gene structures and intron-exon sizes are comparable to the model plants Arabidopsis and rice. However, higher levels of transcriptional complexity in animals with prevalence of alternate splicing and overlapping antisense transcripts need to be properly accounted for. Genomes of higher mammals pose additional difficulties due to the presence of short exons separated by large intronic regions and low-complexity transposon-related sequences in their gene structures which spawn smRNAs.

### The biological significance of antisense ncRNAs

The congruence of antisense tiling array signals to the exonic regions [14] manifest in antisense S/N ratios >>1 for most annotated genes (Supplemental Figure S3, Datafile S4) is remarkable and strongly suggests that the majority of antisense transcription occurs predominantly on spliced mRNAs and is dependent on the activity of RNA-dependent RNA polymerases. A recent report suggests nuclear RNA distorts transcriptome microarray results, consistent with our inference that cytoplasmic RNA is a major source of antisense RNAs [141]. However, deep sequencing of smRNAs from Arabidopsis does uncover a small percentage that map to intron-exon junctions and introns, suggesting that precursor-mRNAs, or more likely DNA in the nucleus is also a source of antisense transcription [78].

Expression of ncRNAs is commonly regulated by stress and environmental stimuli, and many different ncRNAs accumulate at specific developmental stages or in specific cell types, or even within specific subcellular domains, suggesting important and tightly controlled biological roles [1], [16], [25], [56]. New miRNAs continue to be discovered by deep sequencing and are expressed at very low levels or only in a few tissues or at particular times during development. It is speculated that the antisense miRNA* signal we observe (Figure 2) is due to hybridization of pre-miRNA transcripts, but other interpretations are possible such as spurious labeling of abundant miRNA and miRNA* species, or hybridization of miRNA target mRNAs, or mismatch hybridization of homologues. Analysis of available whole tiling array data RNAi knockdown mutants of the exosome [116] and other miRNA metabolism and RNA processing mutants such as *xrn4/abh1* double mutant, *hyponastic leaves1*, *argonaute1*, nonsense-mediated decay effectors *upf1/upf3*, *RNA-dependent RNA polymerase2* (*rdr2*), DNA methylation triple mutant *drm1/drm2/cmt3*, and *serrate* [114], [142]–[145] should be informative, especially when combined with machine learning to find other affected loci.

There remains the important question of the biological significance of antisense transcription as it relates to our findings and the myriad examples found across eukaryotic phyla. It has been postulated that evolution of *MIRNA* genes includes an early stage when antisense transcription is triggered by long perfect dsRNA of an inverted repeat or transposon-related repeat [77], [78], [111], [112]. Our finding of a long inverted repeat with abundant 21 and 24 n.t. siRNAs mapping within antisense ncRNA (Group4327) highly homologous to *MIRNA843* hairpin (Table 3) yet not conserved for mature miR843 (data not shown) suggests a different evolutionary origin of *MIRNA843* than postulated by Fahlgren et al. [77] who noted weak foldback homology with a protein-coding gene (At3g48340) not targeted by miR843. Recent findings in pollen and female gametes and their accessory cells [146], [147], endosperm [148]–[150] and gametes of mouse [151] and Drosophila [123], [152] show endogenous siRNAs are formed from *cis* and *trans* antisense transcripts and function in epigenetic regulation of germ line gene expression and cell fate and may serve a ‘memory’ role to mediate RNA- and DNA level silencing of transposons during vertical transmission to the next generation. A link between genomic imprinting and RNA silencing in plants has come from studies of PolIVb/V-dependent siRNA accumulation in the maternal gametophyte and developing seed: expression of siRNAs in endosperm is specifically from maternal chromosomes [150]. Newly discovered gypsy and copia-like retroelements can transpose in hybrid *met1*/wild type epigenomes and in mutants of the chromatin-remodeling ATPase *decrease in dna methylation1* (*ddm1*); subsequent movements are suppressed by RNA-directed DNA methylation that requires Pol IVb,/V and the histone methyltransferase KRYPTONITE (KYP). These results establish that epigenetic control of retrotransposons extends beyond transcriptional suppression [153], [154]. The transposon- and TAS-like hairpins we describe (Table 3, Supplemental Figure S5) which produce phased siRNAs may be cases of post-transcriptional antisense regulation of relevance to protein-coding gene regulation or miRNA evolution [155]. We speculate that similar mechanisms may affect *MIRNA* genes and miRNA targets that produce smRNAs and are subject to DNA methylation [5], [156], [157].

## Supporting Information

Figure S1 *C. elegans* primary (5′ mono-) and secondary (5′-tri-phosphorylated) antisense siRNAs [98] that map to various positions of miRNA hairpins. Primary siRNAs map predominantly to miRNA* positions, and secondary siRNAs map predominantly to loop regions, similar to results seen in Arabidopsis and rice (Figure 1). See Datafile S6 for details.
(0.60 MB TIF)

Figure S2 Comparison of Arabidopsis and rice sense strand signal profiles for highly conserved domains of eight select ribosomal genes, from whole genome tiling arrays. Signal to noise (S/N) ratios were calculated from the arithmetic means of probe signals mapping to exons divided by intron probe signals. For Arabidopsis, signal line colors indicate RNA samples from T87 callus cultures (blue)[82]; flowers (green); root (magenta); light-grown leaves (brown); and suspension cells (tan)[83]. Exons are denoted below the plot as green boxes on the Watson (upper) or Crick (lower) strands (x-axis). Note the trend for increasing signal strengths towards the 3′ end of the gene (arrows) including 3′ UTRs (ovals), especially for rice data, consistent with degradome studies [80], [114].
(1.32 MB TIF)

Figure S3 Antisense strand signal profiles from Arabidopsis whole tiling arrays for eight ribosomal genes of Supplemental Figure S2. Signal to noise (S/N) ratios were calculated from the arithmetic means of probe signals mapping to exons divided by intron probe signals. Signal line colors indicate RNA samples from T87 callus cultures (blue) [82]; flowers (green); root (magenta); light-grown leaves (brown); and suspension cells (tan) [83]. Exons are denoted below the plot as green boxes on the Watson (upper) or Crick (lower) strands (x-axis). Note the antisense signals are largely congruent with exons, suggesting that antisense transcription occurs on mature mRNAs.
(0.85 MB TIF)

Figure S4 Meta-analysis of two exclusive sets of “unknown” annotated proteins (filled diamonds [27] and open circles [Y. Xiao and C.D. Town, personal communication]) plotted as functions of TAIR9 expression quality (y axis) and ratio of sense exon/antisense exon expression (data from [82], [83]). All genes have independent evidence of antisense expression [58]. The average expression ratios for all 105 genes correlated positively (r = 0.61) as a function of expression rating class, whereas there was an inverse correlation (r = 0.83) between expression rating classes and numbers of genes with sense/antisense expression ratios <1.
(0.72 MB TIF)

Figure S5 (A) Hairpin-containing secondary structure corresponding to phased antisense siRNAs mapping to predicted small ORF At1g55045. Base-pair probabilities from RNAfold [89] are shown as heat map. (B) Phased siRNAs [94] to At1g55045 hairpin mapped with pssRNAMiner [158], P<6e-5 (random hypergeometric distribution). Antisense strand is labeled (-). Approximately 20% of all known smRNAs mapping to this locus are phased.
(2.37 MB TIF)

Table S1 Antisense transcription signals relative to sense strand expression from rice whole genome tiling arrays.
(0.05 MB DOC)

Table S2 List of unknown protein-coding genes with antisense ESTs and abundant antisense transcription from whole genome tiling array data, suggesting mis-annotation of ncRNAs.
(0.19 MB DOC)

Text File S1 Assessment of whole genome tiling array data quality by ribosomal gene expression.
(0.03 MB DOC)

Datafile S1 (0.35 MB XLS)

Datafile S2 (7.22 MB XLS)

Datafile S3 (0.89 MB XLS)

Datafile S4 (8.56 MB XLS)

Datafile S5 (5.88 MB ZIP)

Datafile S6 (0.07 MB XLS)
