# Supplementary figures and images for: Analysis of Antisense Expression by Whole Genome Tiling Microarrays and siRNAs Suggests Mis-Annotation of Arabidopsis Orphan Protein-Coding Genes
Source: PLoS One. 2010 May 26;5(5):e10710. doi: 10.1371/journal.pone.0010710 (PMC2877095; doi:10.1371/journal.pone.0010710)

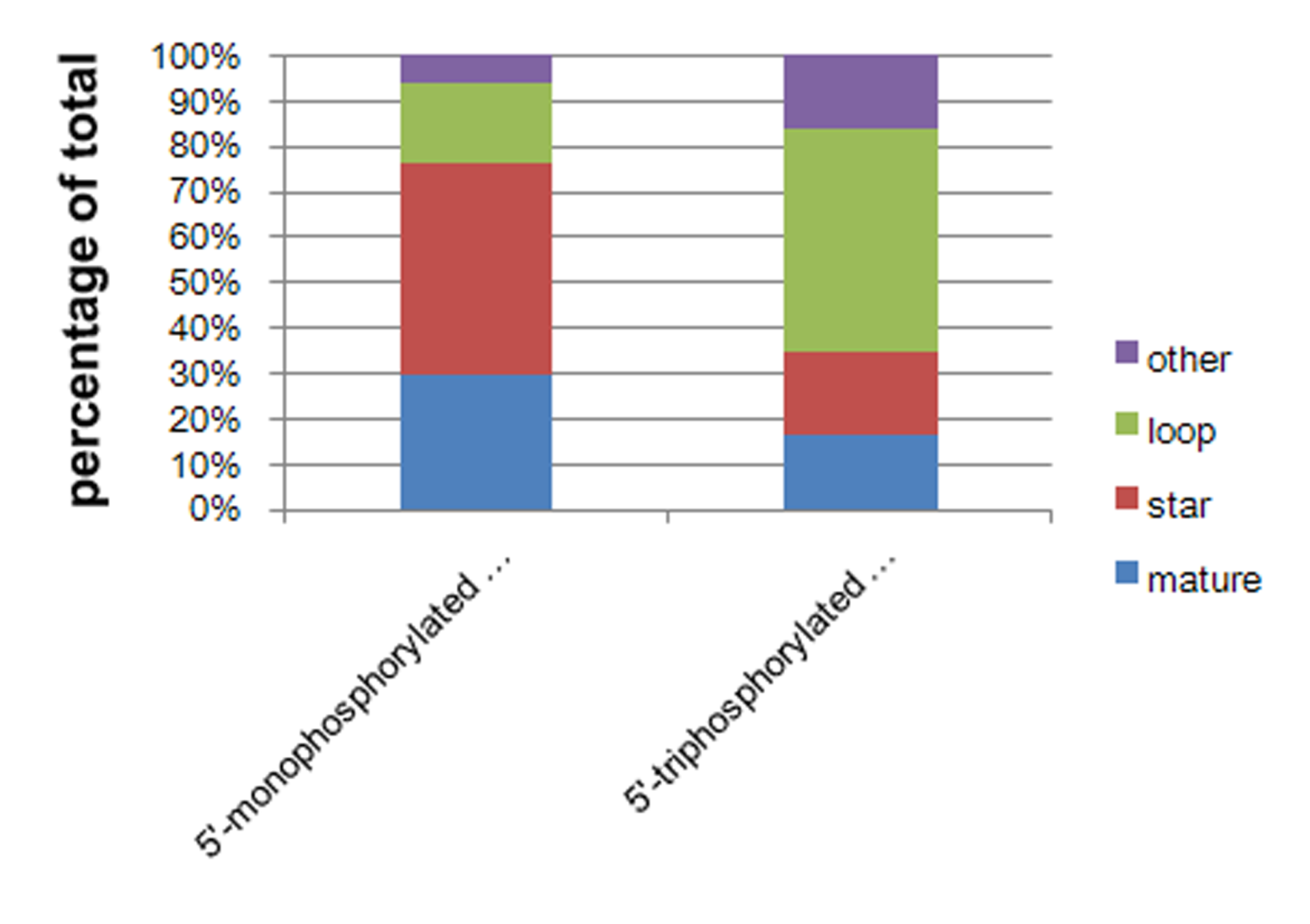

Supplement: Figure S1 — C. elegans primary (5′ mono-) and secondary (5′-tri-phosphorylated) antisense siRNAs [98] that map to various positions of miRNA hairpins. Primary siRNAs map predominantly to miRNA* positions, and secondary siRNAs map predominantly to loop regions, similar to results seen in Arabidopsis and rice (Figure 1). See Datafile S6 for details. (0.60 MB TIF) [file pone.0010710.s001.tif]

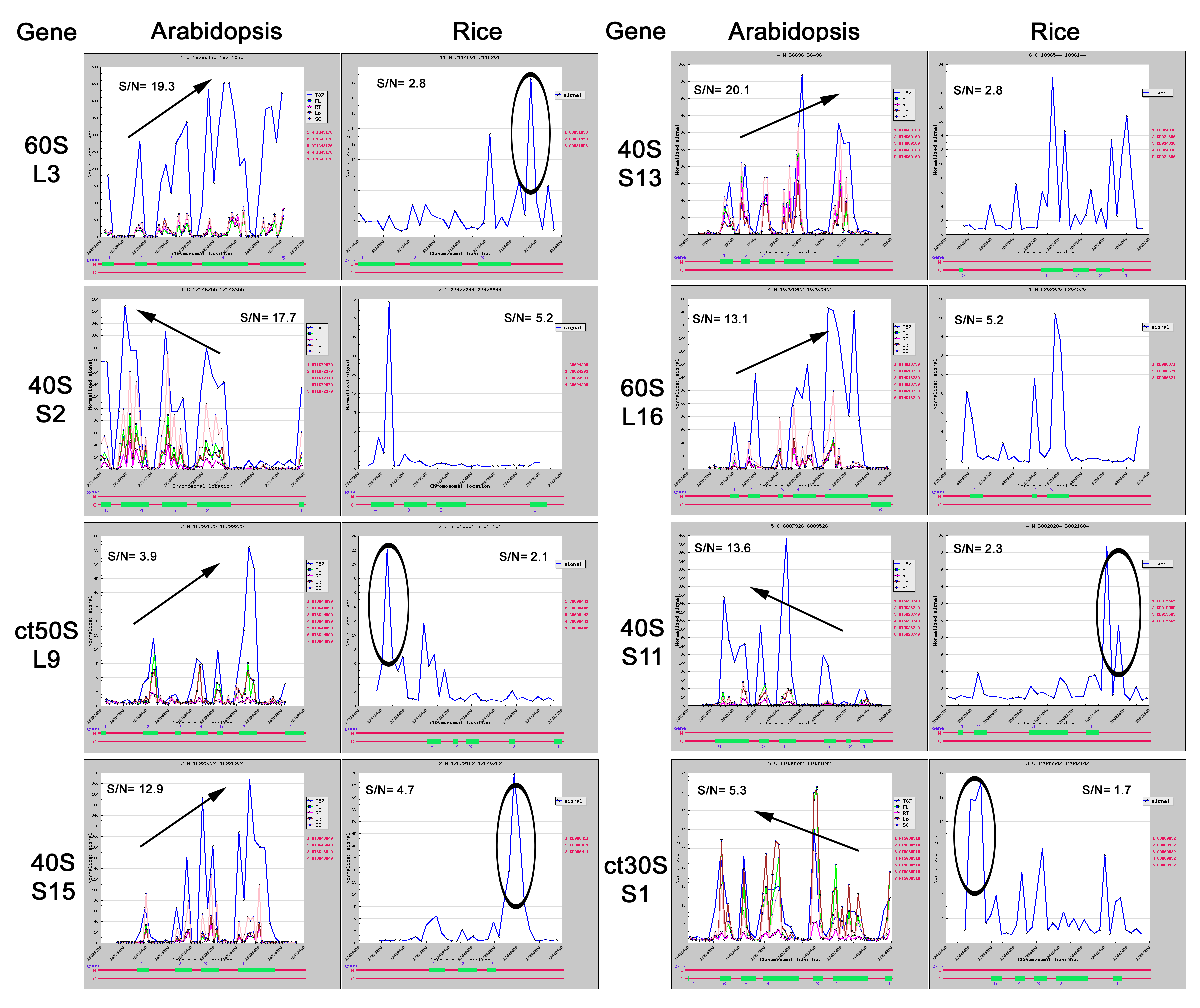

Supplement: Figure S2 — Comparison of Arabidopsis and rice sense strand signal profiles for highly conserved domains of eight select ribosomal genes, from whole genome tiling arrays. Signal to noise (S/N) ratios were calculated from the arithmetic means of probe signals mapping to exons divided by intron probe signals. For Arabidopsis, signal line colors indicate RNA samples from T87 callus cultures (blue)[82]; flowers (green); root (magenta); light-grown leaves (brown); and suspension cells (tan)[83]. Exons are denoted below the plot as green boxes on the Watson (upper) or Crick (lower) strands (x-axis). Note the trend for increasing signal strengths towards the 3′ end of the gene (arrows) including 3′ UTRs (ovals), especially for rice data, consistent with degradome studies [80], [114]. (1.32 MB TIF) [file pone.0010710.s002.tif]

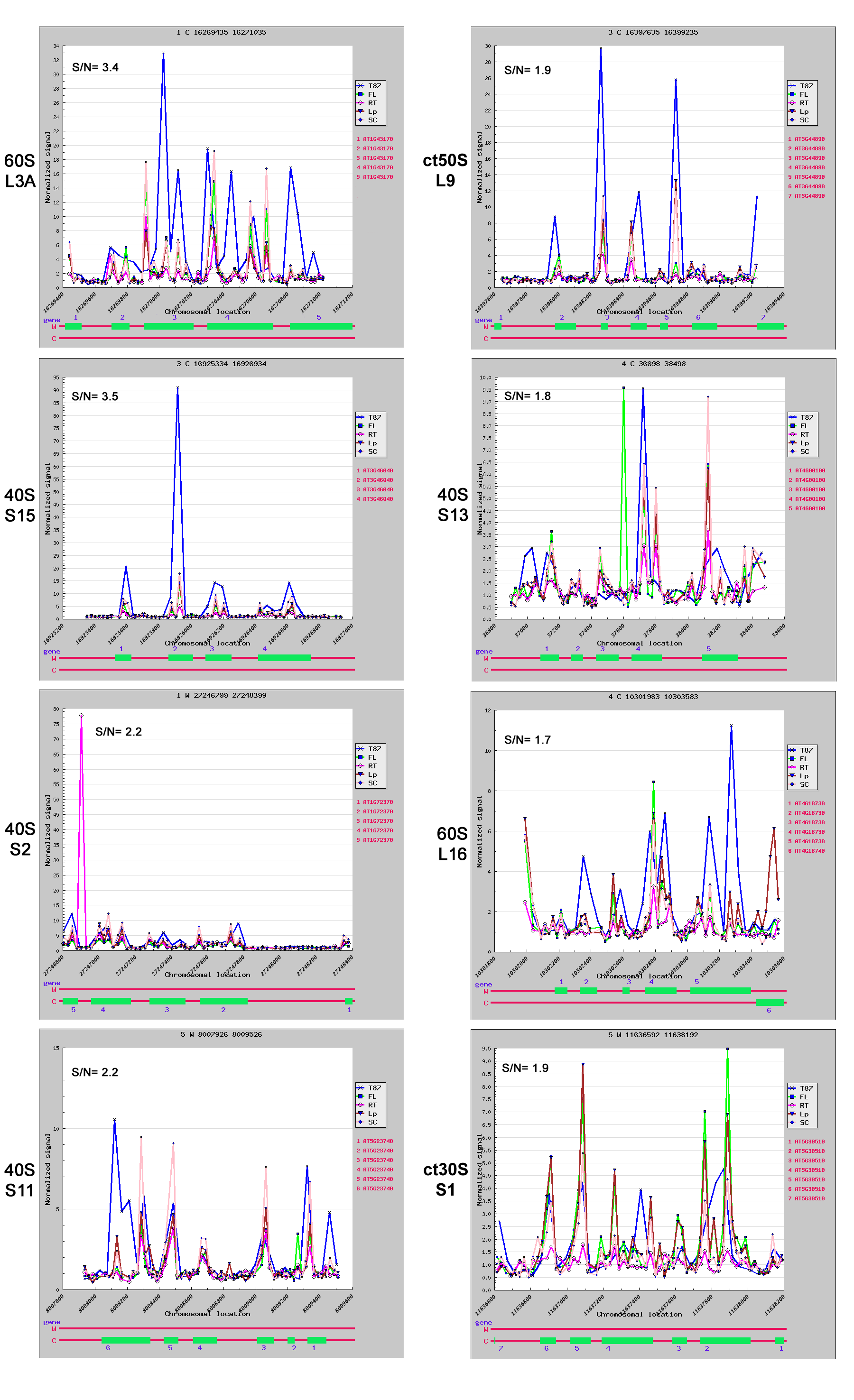

Supplement: Figure S3 — Antisense strand signal profiles from Arabidopsis whole tiling arrays for eight ribosomal genes of Supplemental Figure S2. Signal to noise (S/N) ratios were calculated from the arithmetic means of probe signals mapping to exons divided by intron probe signals. Signal line colors indicate RNA samples from T87 callus cultures (blue) [82]; flowers (green); root (magenta); light-grown leaves (brown); and suspension cells (tan) [83]. Exons are denoted below the plot as green boxes on the Watson (upper) or Crick (lower) strands (x-axis). Note the antisense signals are largely congruent with exons, suggesting that antisense transcription occurs on mature mRNAs. (0.85 MB TIF) [file pone.0010710.s003.tif]

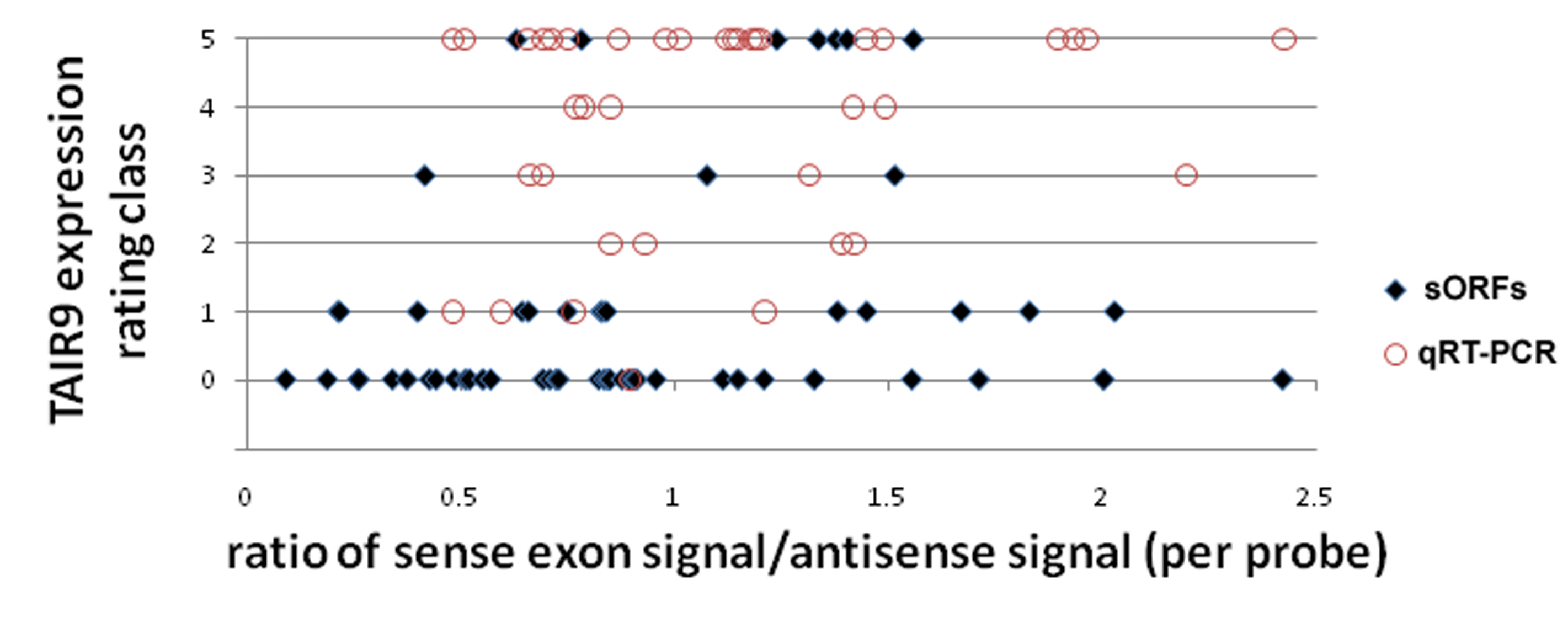

Supplement: Figure S4 — Meta-analysis of two exclusive sets of “unknown” annotated proteins (filled diamonds [27] and open circles [Y. Xiao and C.D. Town, personal communication]) plotted as functions of TAIR9 expression quality (y axis) and ratio of sense exon/antisense exon expression (data from [82], [83]). All genes have independent evidence of antisense expression [58]. The average expression ratios for all 105 genes correlated positively (r = 0.61) as a function of expression rating class, whereas there was an inverse correlation (r = 0.83) between expression rating classes and numbers of genes with sense/antisense expression ratios <1. (0.72 MB TIF) [file pone.0010710.s004.tif]

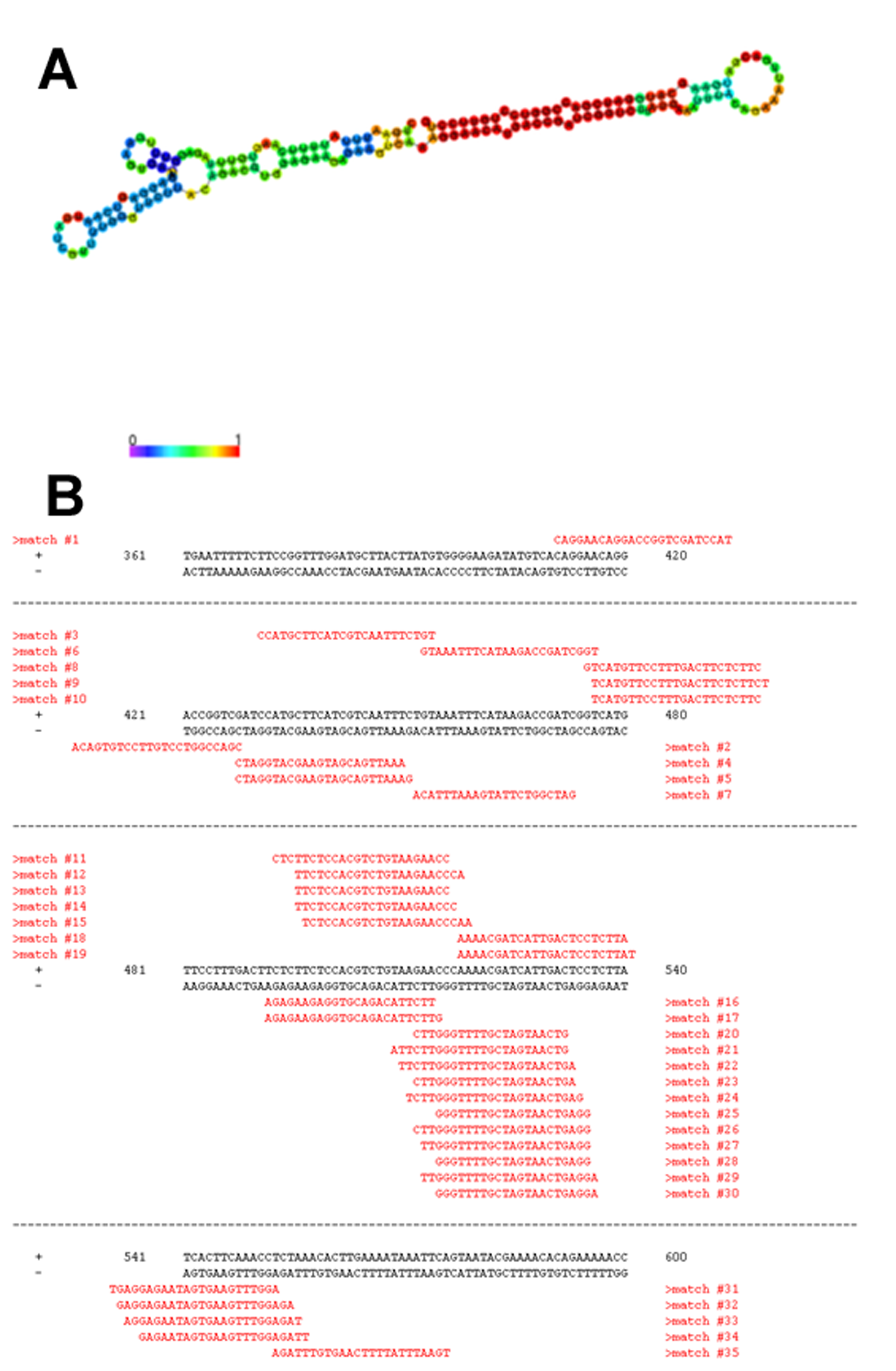

Supplement: Figure S5 — (A) Hairpin-containing secondary structure corresponding to phased antisense siRNAs mapping to predicted small ORF At1g55045. Base-pair probabilities from RNAfold [89] are shown as heat map. (B) Phased siRNAs [94] to At1g55045 hairpin mapped with pssRNAMiner [158], P<6e-5 (random hypergeometric distribution). Antisense strand is labeled (-). Approximately 20% of all known smRNAs mapping to this locus are phased. (2.37 MB TIF) [file pone.0010710.s005.tif]
