# Supplementary material for: Analysis of Antisense Expression by Whole Genome Tiling Microarrays and siRNAs Suggests Mis-Annotation of Arabidopsis Orphan Protein-Coding Genes
Source: PLoS One. 2010 May 26;5(5):e10710. doi: 10.1371/journal.pone.0010710 (PMC2877095; doi:10.1371/journal.pone.0010710)
Supplement: Table S1 — Antisense transcription signals relative to sense strand expression from rice whole genome tiling arrays. (0.05 MB DOC) [file pone.0010710.s006.doc]

**Table S1.** Antisense transcription signals relative to sense strand expression from rice whole genome tiling arrays^a^

| **Gene class** | **Genes with low sense/antisense exon signal ratio** | **Genes with high sense/antisense exon signal ratio** | **Ratio** | ***P* value^b^** |
| --- | --- | --- | --- | --- |
| **Ribosomal gene** | 108 | 296 | 0.36 | 0.000001 |
| **miRNA target^c^** | 20 | 22 | 0.91 | 0.44 |
| **“unknown” gene** | 76 | 57 | 1.33 | 0.06 |
| **expressed gene** | 3065 | 2955 | 1.04 | 0.08 |
| **hypothetical gene** | 4420 | 4845 | 0.91 | 0.000005 |
| **All “unknown, expressed and hypothetical” genes with antisense smRNAs** | 1342 | 1415 | 0.95 | 0.09 |

^a^: Rice gene annotation was from Rice Annotation Release 6.1 [105]. Rice whole genome tiling array data was from [84]. For each gene, the ratio of sense/antisense exon signal is calculated according to the following formula: ratio = [(sense exon signals/probe numbers)/(antiense exon signals/probe numbers)] / [(sense intron signals/probe numbers)/(antiense intron signals/probe numbers)]. See “Material and Method” and Supplemental Datafile 5 for details.

^b^: One-tailed binomial distribution, normal approximation model

^c^: predicted targets for rice miRs 156, 162, 168, 172, and 393. These targets produce significant numbers of antisense siRNAs in Arabidopsis [14]. Validated targets for

miRs 444, 809, 818, 820 and 1425 are also included [100-102].
