# Supplementary material for: Analysis of Antisense Expression by Whole Genome Tiling Microarrays and siRNAs Suggests Mis-Annotation of Arabidopsis Orphan Protein-Coding Genes
Source: PLoS One. 2010 May 26;5(5):e10710. doi: 10.1371/journal.pone.0010710 (PMC2877095; doi:10.1371/journal.pone.0010710)
Supplement: Table S2 — List of unknown protein-coding genes with antisense ESTs and abundant antisense transcription from whole genome tiling array data, suggesting mis-annotation of ncRNAs. (0.19 MB DOC) [file pone.0010710.s007.doc]

**Table S2.** List of unknown protein-coding genes with antisense ESTs and abundant antisense transcription from whole genome tiling array data, suggesting mis-annotation of ncRNAs

| **AGI name** | **Star Overall Rating** | **average exon sense/anti/**  **probe** | **Subset of unknown annotated genes^a^** | **GenBank antisense ESTs** | **Additionalnotes** |
| --- | --- | --- | --- | --- | --- |
| AT1G04778 | 5 | 0.34 | Group144 | DQ49220, DQ652598 | clustered |
| AT1G07901 | 1 | 0.66 | Group247 | BE039024, DR352651, DR352652, DR381128 |  |
| AT1G41875 | 1 | 0.95 | smORF | CF652025 | clustered |
| AT1G48145 | 1 | 0.63 | TIGR | EG457224 | clustered |
| AT1G48730 | 1 | 0.62 |  | AK175404 | as- siRNAs  clustered |
| AT1G53541 | 1 | 0.47 | smORF | EG420033, EL329214, EL320311, AU236017 |  |
| AT2G05915 | 1 | 0.37 |  | DR262957 et al. | as- siRNAs, clustered |
| AT2G07825 | 5 | 0.63 | Group2185 | EG425724 |  |
| AT2G11623 | 1 | 0.43 |  | AY500343, DQ069809 | clustered |
| AT2G13422 | 1 | 0.28 | smORF | AK229194, AU237685, BX839943 | clustered |
| AT2G13547 | 1 | 0.96 | smORF | BT025680 | clustered |
| AT2G16019 | 1 | 0.34 | smORF | EG432832, EG432831, EG432831 |  |
| AT2G45406 | 3 | 0.53 | Group3215 | DR353195 |  |
| AT3G01516 | 5 | 0.49 | Group3332 | AV531480 |  |
| AT3G15604 | 1 | 0.36 | smORF | EG490219, EG490222, EG493861 | clustered |
| AT3G25719 | 1 | 0.32 | smORF | EG429897 |  |
| AT3G28420 | 5 | 0.45 | Group4132 | AI997454 |  |
| AT3G28918 | 1 | 0.65 | Group4143 | EG478300, EG478313 | clustered |
| AT3G43291 | 1 | 0.64 | smORF | EG431723 |  |
| AT3G48298 | 1 | 0.81 | smORF | ES187722 | clustered |
| AT3G49551 | 1 | 0.27 | smORF | BX824305, BX823204, AK176168 | clustered |
| AT4G01985 | 3 | 0.19 | Group4869 | AI995552, AU227942 | clustered |
| AT4G04078 | 1 | 0.74 | smORF | DR381718, DR287670, AU230267, AU239005 | clustered |
| AT4G09649 | 1 | 0.40 | Group5056 | DR195358 | clustered |
| AT4G11300 | 5 | 0.38 | Group5091 | AK221563 |  |
| AT4G28068 | 1 | 0.53 | smORF | DR242858, DR242848 | clustered |
| AT4G36791 | 1 | 0.93 | smORF | AK228751 |  |
| AT5G11425 | 1 | 0.47 | smORF | EG491292, EG455039 | clustered |
| AT5G40595 | 1 | 0.59 |  | DQ132734 | clustered |
| AT5G49440 | 5 | 0.17 | Group7169 | U65471 | as- siRNAs |
| AT5G50562 | 5 | 0.41 | smORF | EL155153, EL182558, BP854112, BP866447, BP851086 | clustered |
| AT5G57567 | 5 | 0.43 |  | DQ652813 |  |
| AT5G66053 | 5 | 0.59 | smORF | AY045912 | clustered |
| AT1G05730 | 5 | 0.87 | Group167 | nat-cis-as with AT1G05720 |  |
| AT1G20290 | 1 | 0.60 |  | nat-cis-as with At1g20300 | as- siRNAs |
| AT1G24822 | 1 | 0.23 |  | nat-cis-as with ncRNA At1g24825 | as- siRNAs  clustered |
| AT1G24996 | 1 | 0.13 |  | nat-cis-as with At1g25054 |  |
| AT1G25097 | 1 | 0.22 |  | nat-cis-as with ncRNA At1g25098 | as- siRNAs  clustered |
| AT1G27640 | 5 | 0.27 |  | nat-cis-as with At1g27650 |  |
| AT1G29355 | 1 | 0.60 | Group836 | nat-cis-as with At1g29357 |  |
| AT1G29680 | 5 | 0.60 | Group848 | nat-cis-as with At1g29670 |  |
| AT1G44780 | 5 | 0.58 | Group1055 | nat-cis-as with At1g44790 |  |
| AT1G45545 | 3 | 0.41 |  | nat-cis-as with At1g45474 | clustered |
| AT1G52325 | 1 | 0.69 | Group1184 | nat-cis-as with AT1G52320 |  |
| AT1G62240 | 5 | 0.41 |  | nat-cis-as with At1g62250 |  |
| AT1G67790 | 1 | 0.56 |  | nat-cis-as with ncRNA At1g67792; AK226625 |  |
| AT1G68700 | 4 | 0.58 | Group1572 | nat-cis-as with At1g68710 |  |
| AT1G71760 | 4 | 0.51 | TIGR | nat-cis-as with AT1G71770 |  |
| AT1G76780 | 1 | 0.40 | Group1833 | nat-cis-as with At1g76790 | clustered |
| AT2G04380 | 1 | 0.67 | Group2072 | nat-cis-as with At2g04378 | clustered |
| AT2G07215 | 1 | 0.62 | TIGR | nat-cis-as with ncRNA At2g07213 | clustered |
| AT2G10920 | 5 | 0.23 |  | nat-cis-as with ncRNA At2g10921 | as- siRNAs  clustered |
| AT2G13430 | 1 | 0.53 |  | nat-cis-as with ncRNA At2g13431 | as- siRNAs  clustered |
| AT2G18270 | 5 | 0.36 |  | nat-cis-as with At2g18280 | clustered |
| AT2G22088 | 1 | 0.51 |  | nat-cis-as with At2g22090 |  |
| AT2G22820 | 5 | 0.38 | TIGR | nat-cis-as with ncRNA At2g22821 | clustered |
| AT2G30760 | 1 | 0.62 | Group2713 | nat-cis-as with At2g30766 | clustered |
| AT2G30960 | 4 | 0.39 |  | nat-cis-as with At2g30950 |  |
| AT2G34120 | 0 | 0.14 |  | nat-cis-as with At2g34100 | clustered |
| AT2G36030 | 5 | 0.28 |  | nat-cis-as with At2g36026 |  |
| AT2G36355 | 5 | 0.28 |  | nat-cis-as with At2g36360 |  |
| AT2G39520 | 1 | 0.45 |  | nat-cis-as with At2g39518 | clustered |
| AT2G39975 | 1 | 0.49 |  | nat-cis-as with At2g39980; DQ069850 | clustered |
| AT2G42370 | 1 | 0.62 | Group3112 | nat-cis-as with At2g42365 |  |
| AT2G43795 | 4 | 0.59 | Group3166 | nat-cis-as with At2g43790 | clustered |
| AT3G15550 | 4 | 0.49 | Group3779 | nat-cis-as with At3g15570 |  |
| AT3G18540 | 1 | 0.19 | Group3872 | nat-cis-as with ncRNA At3g18535 | as- siRNAs |
| AT3G27415 | 3 | 0.42 |  | nat-cis-as with At3g27416 |  |
| AT3G46666 | 5 | 0.69 | Group4292 | nat-cis-as with ncRNA At3g46668 | clustered |
| AT3G53235 | 1 | 0.21 |  | nat-cis-as with At3g53240 | clustered |
| AT4G01670 | 4 | 0.35 |  | nat-cis-as with At4g01660 | as- siRNAs |
| AT4G03940 | 1 | 0.54 |  | nat-cis-as with ncRNA At4g03935 | clustered |
| AT4G13320 | 5 | 0.63 | Group5145 | nat-cis-as with At4g13330; BX827932 |  |
| AT4G18500 | 1 | 0.19 |  | nat-cis-as with At4g18501 | clustered |
| AT4G20290 | 5 | 0.43 |  | nat-cis-as with At4g20300 |  |
| AT4G21930 | 5 | 0.58 | Group5402 | nat-cis-as with At4g21926 |  |
| AT4G22510 | 1 | 0.51 | Group5417 | nat-cis-as with At4g22500 and At4g22520 | clustered |
| AT4G24030 | 1 | 0.31 | Group5456 | nat-cis-as with At4g24026 |  |
| AT4G26288 | 5 | 0.61 | Group5538 | nat-cis- as with At4g26290 and At4g26300 |  |
| AT4G26290 | 5 | 0.60 | Group5538 | nat-cis-as with At4g26288; BX823434 | clustered |
| AT4G37409 | 4 | 0.46 | Group5899 | nat-cis-as with At4g37410 |  |
| AT5G02720 | 5 | 0.47 |  | nat-cis-as with At5g02710 | clustered |
| AT5G14560 | 5 | 0.29 |  | nat-cis-as with At5g14550 |  |
| AT5G16160 | 5 | 0.31 |  | nat-cis-as with At5g16150 | clustered |
| AT5G26840 | 1 | 0.68 |  | nat-cis-as with At5g26830 | as- siRNAs clustered |
| AT5G27020 | 5 | 0.39 |  | nat-cis-as with At5g27030 |  |
| AT5G28920 | 1 | 0.68 | Group6779 | nat-cis-as with At5g28919 | as- siRNAs  clustered |
| AT5G41140 | 4 | 0.51 | Group6938 | nat-cis-as with At5g41150 |  |
| AT5G41320 | 1 | 0.36 |  | nat-cis-as with At5g41330 |  |
| AT5G53895 | 4 | 0.32 |  | nat-cis-as with At5g53900 | clustered |
| AT5G55570 | 5 | 0.44 | Group7351 | nat-cis-as with At5g55580 | clustered |
| AT5G57310 | 1 | 0.50 |  | nat-cis-as with At5g57300 |  |
| AT5G63340 | 1 | 0.66 |  | nat-cis-as with At5g63320 | clustered |
| AT5G64820 | 5 | 0.44 |  | nat-cis-as with AT5g64816 |  |
| AT5G64910 | 2 | 0.23 | Group7633 | nat-cis-as with At5g64920 | clustered |
| AT5G65030 | 5 | 0.42 |  | nat-cis-as with At5g65020 |  |
| AT5G65580 | 5 | 0.29 | Group7653 | nat-cis-as with ncRNA At5g65575 | clustered |
| AT5G65610 | 1 | 0.51 |  | nat-cis-as with At5g65609 | as- siRNAs |
| AT2G11891 | 1 | 0.34 | smORF |  | 3' exon |
| AT3G24516 | 5 | 0.33 | smORF |  | 3' exon, clustered |
| AT1G53620 | 5 | 0.36 | TIGR |  | 3' exon |
| AT4G03505 | 5 | 0.61 | Group4926 |  | 3' exon, clustered |
| AT4G37095 | 2 | 1.04 | smORF |  | 3' exon, clustered |
| AT3G24517 | 1 | 0.54 |  |  | 3' exon of ncRNA At3g24518 |
| AT3G15909 | 1 | 0.78 | smORF |  | 5' exon, clustered |
| AT2G34238 | 1 | 0.49 | smORF |  | 5' exon, clustered |
| AT2G34224 | 1 | 0.62 | smORF |  | 5' exon, clustered |
| AT2G14289 | 1 | 0.91 | smORF |  | 5' exon, clustered |
| AT2G11651 | 1 | 0.79 | smORF |  | 5' exon, clustered |

^a^: ”Group” genes are previously described antisense ncRNAs [58]. “SmORFs” are those genes described by Hanada et al. [27]. “TIGR” genes are those annotated in TAIR9 (Y. Xiao and C.D. Town, personal communication).
