# Supplementary material for: Analysis of Antisense Expression by Whole Genome Tiling Microarrays and siRNAs Suggests Mis-Annotation of Arabidopsis Orphan Protein-Coding Genes
Source: PLoS One. 2010 May 26;5(5):e10710. doi: 10.1371/journal.pone.0010710 (PMC2877095; doi:10.1371/journal.pone.0010710)
Supplement: Text File S1 — Assessment of whole genome tiling array data quality by ribosomal gene expression. (0.03 MB DOC) [file pone.0010710.s008.doc]

**Supplemental Text**

Assessment of whole genome tiling array data quality by ribosomal gene expression

Based on the marginal signals observed for rice *MIRNA* gene expression probed by whole genome tiling arrays, we sought to qualify the rice tiling array dataset by objective criteria, in the first instance sense strand expression of ribosomal genes which are deeply conserved and highly expressed across eukaryotes. We chose several rice nuclear-encoded ribosomal protein genes for large and small subunits of cytosolic- and chloroplast-localized ribosomes based on high sequence homology with Arabidopsis, and manually calculated signal-to-noise (S/N) ratios by averaging probe signals for exonic (signal) and intronic (noise) probes. Figure S2 shows side-by-side graphical comparisons between Arabidopsis and rice whole tiling array expression signals for portions of eight different ribosomal protein gene homologues. Arabidopsis tiling array expression data typically had cumulative S/N > 10 for all samples, but for the sole rice sample it was much lower, on the order of S/N ~3 with the majority of signals mapping to the 3’ UTR (Fig. S2, ovals) which was not included in the S/N formula. In stark contrast, Arabidopsis signals showed excellent congruence with exon/intron annotations in all five samples (from two different technology platforms), with only a slight bias of expression signal toward the 3’ end of the gene (arrows) and little signal abundance in the 3’-UTRs (Fig. S2). Taken together, these results indicate a high quality transcriptome dataset for Arabidopsis, but not for rice. Next the antisense strand for these same genes was manually analyzed and the result for Arabidopsis is shown in Supplemental Figure S3. The S/N averaged about 2 for Arabidopsis the antisense strand, with a remarkable congruence of signal to the exonic regions, consistent with the model [78] that antisense transcription occurs predominantly on spliced mRNAs. In contrast, for rice the antisense strand S/N was several-fold worse than for Arabidopsis (data not shown), similar to that seen for the sense strand (Fig. S2).
